# Study of the Rv1417 and Rv2617c Membrane Proteins and Their Interactions with Nicotine Derivatives as Potential Inhibitors of Erp Virulence-Associated Factor in *Mycobacterium tuberculosis*: An In Silico Approach

**DOI:** 10.3390/biom13020248

**Published:** 2023-01-28

**Authors:** Jorge Alberto Aguilar-Pineda, Camilo Febres-Molina, Cinthia C. Cordova-Barrios, Lizbeth M. Campos-Olazával, Bruno A. Del-Carpio-Martinez, Flor Ayqui-Cueva, Pamela L. Gamero-Begazo, Badhin Gómez

**Affiliations:** 1 Centro de Investigación en Ingeniería Molecular—CIIM, Universidad Católica de Santa María, Urb. San José s/n, Umacollo, Arequipa 04013, Peru; 2 Doctorado en Fisicoquímica Molecular, Facultad de Ciencias Exactas, Universidad Andres Bello, Santiago de Chile 8370134, Chile; 3 Departamento de Ciencias Farmacéuticas, Bioquímicas y Biotecnológicas, Universidad Católica de Santa María, Urb. San José s/n, Umacollo, Arequipa 04013, Peru; 4 Facultad de Arquitectura e Ingeniería Civil y del Ambiente, Universidad Católica de Santa María, Urb. San José s/n, Umacollo, Arequipa 04013, Peru

**Keywords:** tuberculosis, Erp, Rv1417, Rv2617c, nicotine analogs, molecular dynamics

## Abstract

The increasing emergence of *Mycobacterium tuberculosis* (Mtb) strains resistant to traditional anti-tuberculosis drugs has alarmed health services worldwide. The search for new therapeutic targets and effective drugs that counteract the virulence and multiplication of Mtb represents a challenge for the scientific community. Several studies have considered the *erp* gene a possible therapeutic target in the last two decades, since its disruption negatively impacts Mtb multiplication. This gene encodes the exported repetitive protein (Erp), which is located in the cell wall of Mtb. In vitro studies have shown that the Erp protein interacts with two putative membrane proteins, Rv1417 and Rv2617c, and the impairment of their interactions can decrease Mtb replication. In this study, we present five nicotine analogs that can inhibit the formation of heterodimers and trimers between these proteins. Through DFT calculations, molecular dynamics, docking, and other advanced in silico techniques, we have analyzed the molecular complexes, and show the effect these compounds have on protein interactions. The results show that four of these analogs can be possible candidates to counteract the pathogenicity of Mtb. This study aims to combine research on the Erp protein as a therapeutic target in the search for new drugs that serve to create new therapies against tuberculosis disease.

## 1. Introduction

*Mycobacterium tuberculosis* (*Mtb*) is the bacterium causative of tuberculosis (*TB*), the second most infectious disease after COVID-19 [1,2,3]. *TB* is spread by airborne microparticles from an infected person, and is able to inhibit the apoptosis of its host, thereby increasing its rate of replication in the body [4,5,6]. Since 1800, *TB* has been the primary cause of health deterioration and even mortality worldwide [7]. However, it was not until the early 1940s that the development of pharmaceuticals, good hygiene, and improved nutritional practices caused the mortality rate to decline by 10% per year [8]. Unfortunately, this disease has not been eradicated yet. In 2020, an estimated 10 million people were infected with *TB* worldwide: 5.6 million men, 3.3 million women, and 1.1 million children [3,9]. Nowadays, several regions of the planet still show an increasing incidence of this disease [10]. Standard treatment consists of administering four first-line drugs for six months with a success rate of 85% (isoniazid, rifampicin, ethambutol, and pyrazinamide for two months, followed by four months of isoniazid and rifampicin) [11,12]. Even so, *Mtb* is able to evade the immune mechanisms of the host organism and survive within its host cell due to its ability to alternate between active and latent states [13,14,15].

Although the incidence of *TB* has declined in recent years, as mentioned above, some WHO regions have not made progress in reducing new cases [3]. An example of this is Latin America, where the incidence rate has only decreased by 1.6% in the last two decades. According to the Pan American Health Organization (PAHO), Haiti, Peru, and Bolivia are the countries with the highest incidence rates, exceeding 100 cases per 100,000 inhabitants [16]. This high incidence is mainly due to inadequate prescription and treatment. In addition, poor patient adherence to treatment hinders the diagnosis and, therefore, the adequate treatment of this disease [3,17]. Non-compliance with treatment has allowed *Mtb* to develop a high capacity for resistance to anti-tuberculosis agents. Drug-resistant (DR–TB), multidrug-resistant (MDR–TB), rifampicin-resistant (RR–TB), and extremely drug-resistant (XDR–TB) are just some examples of new variants of *Mtb* [18,19,20]. There are drugs currently approved by the U.S. Food and Drug Administration (FDA), such as bedaquiline, delamanid, and pretomanid [21]; by 2020, there were 22 drugs in Phase I, II, or III trials, including the compounds BTZ–043, delpazolid, GSK–3036656, macozinone, OPC-167832, Q203, SQ109, SPR720, sutezolid, TBAJ-876, TBA-7371, TBI-166, and TBI-223 [22,23]. Nonetheless, the new anti-tuberculosis drugs are more expensive and require longer treatment periods, which generates significant health, social, and economic problems [3,9,16]. Hence, there is a need to develop new, more effective, affordable, and non-toxic therapeutic strategies, especially for drug-resistant tuberculosis.

Several experimental and in silico strategies have been proposed to develop effective drugs for treating *TB* [24,25,26]. However, they all depend on the knowledge of the Mtb–host interaction mechanisms to elucidate possible therapeutic targets [27]. Unfortunately, Mtb has evolved complex and efficient ways to evade the host immune response, which hinders the search for new drugs and effective therapies. In recent years, a large number of genes involved in the Mtb virulence factor have been identified. Forrellad et al. assembled and analyzed information about the main genes or proteins whose irruption led to the loss of pathogenicity or virulence of the Mtb [28]. In that study, the authors point out that more than 5% of the proteins identified in the cell wall are associated with virulence factors. In this sense, Berthet et al. reported that disruption of the *erp* gene, which encodes the exported repetitive protein (Erp), decreased the Mtb virulence factor both in vitro in macrophage cultures and in vivo in mice models [29].

Despite the importance of the Erp protein in the replication and survival of Mtb, its role in the pathogenesis of *TB* is still poorly explored [30]. However, there is a constant increase in studies aimed at developing drugs using *erp* gene as a therapeutic target [31]. Solans et al. proposed the creation of a *TB* vaccine based on the existing MTBVAC vaccine, which is constructed from the mutation of the *phoP* and *fadD26* virulence genes [32]. The new vaccine, called MTBVAC *erp*${}^{-}$, considers a third inactivation, that of the *erp* gene. This vaccine is designed for patients at high risk of immunosuppression. At the same time, some studies have focused on determining the function of the Erp protein through its novel interactions with other proteins [30]. Thus, Klepp et al. searched for Erp-binding proteins using a bacterial two-hybrid system, determining that Erp interacts with two putative membrane proteins, Rv1417 and Rv2617c [33]. Furthermore, their results indicated that the formed protein complexes were linked through multiple interactions. In this context, our research group presented an in silico study where we analyzed different possible structural conformations of the Erp–Rv1417 and Erp–Rv2617c heterodimers [34]. In that study, we highlight the importance of considering the electrostatic medium in which the proteins interacted and their influence on their binding energies.

Although scarce, experimental studies show that the Mtb bacterium uses these proteins as survival and replication strategies. Forrellad et al. demonstrated that the Rv2617c and the Erp proteins play an essential role in the mechanism of resistance to the microbicidal action of macrophages (oxidative stress) [35]. Furthermore, using a mutant Mtb strain with altered copies of the *Rv2617c* gene, these authors observed that the Rv2617c protein was required for its replication in a mouse model of tuberculosis. In another study, Olvera et al. showed that the interaction of the KdpF peptide with various proteins, including Rv2617c, decreased the replication of the *Mycobacterium bovis* BCG bacterium [36]. On the other hand, the Rv1417 protein has been found in mutant strains resistant to bedaquiline (BDQ), a drug used to treat MDR/RR tuberculosis [37]. Unfortunately, the implication of these interactions has been poorly explored.

Currently, the development of new anti-*TB* drugs based on nicotine derivatives has increased. The importance of these compounds is due to the fact that, in addition to having antibiotic, anti-inflammatory, and anticancer activity [38,39], several have shown high efficacy in treating TB [38,40,41,42]. Furthermore, recent studies on the pharmacological effects and antibiotic properties of nicotine and its analogs have aroused interest in developing potential anti-tuberculous agents [43]. Notable among these studies is the one carried out by Gandhi et al., who analyzed, with in vitro and in silico techniques, 25 nicotine analogs with anti-addictive properties [44]. Using dihydrofolate reductase (DHFR) as the target protein, the researchers obtained five nicotine analogs with an inhibitory effect on *Mtb* growth. Likewise, in the in silico work of Mardianingrum et al., the authors assessed the interactions of four isoniazid derivatives with the enoyl-acyl carrier protein reductase receptor (InhA) [45]. Their study showed that three of these derivatives had better molecular coupling than isoniazid, suggesting these compounds could have better anti-*TB* activity. Both nicotine and isoniazid analogs have a pyridine ring known to have antimicrobial and anti-tuberculous properties. However, assays in mice showed that nicotinamide, a nicotinic acid derivative, had an antagonistic effect on the action of isoniazid [41]. This fact could lead to developing drugs against Mtb strains resistant to isoniazid. Finally, several studies indicate that pyridine analogs may have activity against MDR–*TB* strains [46,47].

The action of nicotine analog molecules (NAMs) on the interactions between Rv1417, Rv2617c, and Erp proteins has not yet been explored. Therefore, this study aims to evaluate whether five specific NAMs can inhibit the formation of heterodimers between these proteins. These five NAMs have demonstrated anti-mycobacterial activity against the Mtb H37Rv strain in in vitro assays [44]. For this purpose were used several in-silico techniques such as MD simulations, molecular docking, electrostatic potential (ESP) surfaces, and free energy calculations. Moreover, based on pocket conservation and direct solvation techniques, novel interaction sites have been proposed as potential therapeutic targets to decrease the virulence factor of Erp proteins. These results demonstrate that these techniques can be used in a complementary approach to predict potential active sites in protein systems without sufficient experimental information as in this case. We hope that these findings can contribute to the development of new drugs and therapies to fight *TB* disease.

## 2. Computational Details

In a previous study, the stable structures of the Rv1417, Rv2617c (RvPs), and Erp proteins were obtained under the membrane and unanchored conditions by molecular dynamic (MD) simulations. In that study, the influence of the medium on the protein interactions of RvP–Erp heterodimers was explored. Results showed that the RvP–Erp dimeric complexes formed in membrane conditions were energetically more favorable than those formed in semipolar environments. Therefore, for this work, we have used membrane conditions to analyze the inhibitor effect of five nicotine analogs on RvP–Erp interactions. To achieve this, new replicas of the protein–membrane complexes were simulated to obtain stable configurations. The initial three-dimensional structures were retrieved from the AlphaFold database [48,49] with codes AF–P9WIQ7-F1 (Erp) [50], AF–P9WLY1–F1 (Rv1417) [51], and AF–I6XER9-F1 (Rv2617c) [52]. Furthermore, in the case of the RvPs, the minimum energy structures were obtained by free energy landscape (FEL) calculations to construct the protein–drug systems.

### 2.1. Protein–Membrane Complexes

For membrane complexes, only Rv1417 and Rv2617 proteins were considered. RvP structures were embedded in a dipalmitoylphosphatidylcholine (DPPC) bilayer with 512 lipid molecules per complex. All membrane bilayers were constructed using a 128-DPPC bilayer with 64 lipid molecules per layer replicated twice in the *x* and *y* directions to obtain the 512–DPPC membrane. In order to embed the protein structures into membranes, the InflateGRO methodology was used [53].

The minimal structure of the Erp protein was obtained without membrane constraints. Therefore, although it has been inferred that its structure may be anchored to the membrane by residues G253-V273 [54], this fact was not considered for further analyses in this work.

### 2.2. Protein–Membrane–Drug Complexes

Once the minimal structures of the RvPs proteins were obtained, the protein–membrane–drug complexes were constructed via two different methods. In the first, a drug molecule was placed in the pockets of each of the Rv proteins through local molecular docking. For this purpose, the MD trajectories were analyzed using the MDPocket server to obtain those pockets or hot spots with the highest conservation during the MD simulations [55]. Then, using the AutoDock Vina software [56,57], the drug–protein interaction with the best interaction energy was chosen. The second method consisted of solvating the systems with 11 molecules of the drug and randomly placing them using the Gromacs *solvate* tool.

### 2.3. MD Simulations

All MD simulations were performed using the GROMACS 2020.4 software suite [58,59]. The OPLS–AA force field was used together with the DPPC parameters to calculate the different interactions of the atoms [60,61,62]. In addition, explicit TIP4p water molecules were used to solvate the systems [63], and Na${}^{+}$ and Cl${}^{-}$ ions were added to mimic physiological conditions and to obtain an ionic strength of 150 mM with a neutral total net charge. The protein–membrane structures were located in the center of triclinic boxes with a distance of 1.3 nm between the surface of the system and the border of the box on the *z*-axis to ensure no problems with periodic boundary conditions. Once obtained, the molecular complexes were minimized energetically by the steepest-descent algorithm with a maximum of 50,000 steps. Two simulations with position restraints were carried out for the equilibrium phase in the NVT and NPT ensembles. The NVT simulation was performed at 323.15 K using a V-rescale thermostat at 50 ps of trajectory. For the NPT simulation, a temperature of 309.65 K was used with a semi-isotropic pressure coupling. The compressibility factor was 4.5 × 10${}^{-5}$ with a reference pressure of 1.0 bar along the 1.0 ns of the trajectory using a Nosé–Hoover thermostat and a Parrinello–Rahman barostat. The MD production phase was performed without position restraints, and was calculated in the isobaric–isothermal ensemble at 309.65 K with semi-isotropic pressure coupling for 500 ns of trajectory for systems without drug molecules and 100 ns for those systems that included them. In addition, periodic boundary conditions (PBCs) in all directions, a particle mesh Ewald (PME) algorithm for long-range electrostatics with cubic interpolation and a cut-off of 1.2 nm, and a linear constraint solver (LINCS) with all bonds constrained were applied for all MD simulations. For the analyses, all trajectories were saved every 15 ps.

### 2.4. Nicotine Analogs

**Figure 1:**
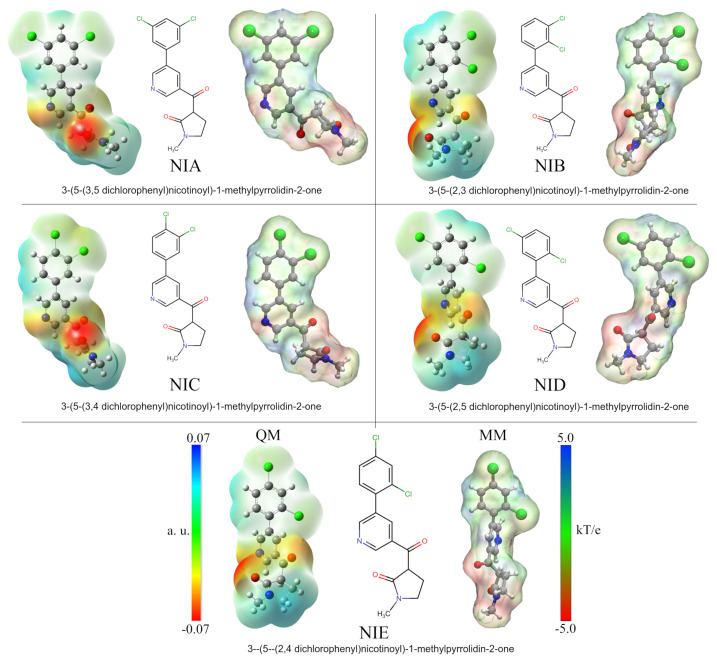
Nicotine analog structures. The 2D depiction shows the different positions of the chlorine substituents. Analog molecules were renamed according to chlorine positions. Optimized structures and quantum ESP surfaces were obtained by DFT calculations using the polarizable continuum model (PCM) with water as an implicit solvent. MM-ESP surfaces were calculated using APBS methodology. On all surfaces, the different colors indicate their molecular electrostatic properties: red for the most nucleophilic zones, dark blue for the most electrophilic zones, and green for neutral.

Five nicotine-derived molecules were considered as ligands in the *Mtb* protein complexes. The molecular properties of these analogs have been described by Gandhi et al. (see Figure 1) [44]. The chemical formulas were obtained from the PubChem server [64], and the 3D structures were constructed using the GaussView v.6 software [65] and optimized by density functional theory (DFT) calculations using the Gaussian 16 software package [66]. The optimization process was performed using the CAM-B3LYP functional [67] and the TZVP basis set [68] with an implicit water solvent effect (*SCRF = SMD; Solvent = Water*). The vibrational frequencies were calculated to ensure that the geometries were in their ground state. Atomic charges with the Hirshfeld population analysis approximation were calculated to consider the electrostatic effects of the ligands in the protein complexes [69,70]. The charges were used to reparametrize the force fields of the nicotine analogs obtained via the LigParGen server [71], which uses OPLS-AA force field parameters to generate them [60,61]. The same atomic charges were used in the construction of molecular ESP surface analyses for the protein–ligand complexes.

### 2.5. Molecular Docking Calculations

Various calculations of molecular dockings were performed for the different analyses presented in this work. As mentioned above, one of them consisted of a local coupling between the different drugs and the hot spots of the Rv1417 and Rv2617c proteins. From our calculations, ten protein–drug complexes were obtained, and the one with the highest binding energy was chosen for the MD simulations. On the other hand, two types of coupling were carried out to evaluate whether the drugs inhibited the interactions between the RVPs and the Erp protein. The first consisted of evaluating complementarity and penetration between protein structures in the presence of the membrane. These calculations were performed using PatchDock software [72,73]. Afterward, the best 1000 structures were subjected to a second docking procedure using FireDock software, which allows the relaxation of the structures and scores them according to their interaction energies [74,75]. From this last calculation, the 100 complexes with the most favorable energies were chosen to analyze contacts between proteins.

### 2.6. *Computation of Binding Free Energy Using MM/PBSA Approximation*

Binding free energies (BFEs) were used to evaluate the protein–protein and protein–ligand affinities. Molecular mechanics Poisson–Boltzmann surface area (MM/PBSA) calculations were performed using the g_mmpbsa methodology [76,77]. The energy contributions per residue were calculated to localize the main interactions and to assess the effect of each residue on the molecular complexes. The last 50 ns of the MD trajectories were analyzed at a regular interval of 0.2 ns to estimate the BFE ($\Delta G_{bind}$), which was calculated using the following equation:

(1) $$\Delta G_{bind}=G_{complex}-\left(G_{Mtb_{prot}}+G_{lig}\right)$$

where $G_{complex}$ is the total free energy of the molecular complexes, and $G_{Mtb_{prot}}$ and $G_{lig}$ are the free energies of isolated *Mtb* proteins and nicotine analogs, respectively. In addition, we used the bootstrap analysis to calculate the average binding energy, which is included in the g_mmpbsa tools. All calculations were obtained at 309.65 K, and default parameters were used to calculate molecular mechanics potential energy and solvation-free energy [77]. Finally, the binding free energy by residue was obtained using

(2) $$\Delta G_{bind}^{res}=\Delta E_{MM}^{res}+G_{p}^{res}+G_{np}^{res}$$

### 2.7. Structure and Data Analysis

Statistical results, root mean squared deviation (RMSD), root mean squared fluctuation (RMSF), radii of gyration (RG), solvent accessible surface area (SASA), hydrogen bonds (HBs), binding free energies (BFEs), structures, trajectories, and B-factor maps were obtained using Gromacs modules. Analysis of structure properties was performed using the MD trajectories of the last 200 ns of each simulation, which were then visualized using Visual Molecular Dynamics (VMD) software [78] and UCSF Chimera v.1.14 [79]. The graphs were plotted using XMGrace software [80]. In addition, 2D representations of electrostatic and hydrophobic interactions were built using LigPlot program [81]. The electrostatic potential surfaces within the molecular mechanics framework (MM-ESP) were calculated in APBS (Adaptive Poisson–Boltzmann Solver) software v.1.4.1 [82], and the pqr entry was created in the PDB2PQR server [83]. Free energy landscape (FEL) maps were used to visualize the energy associated with the protein conformation of the different models during the MD simulations. These maps are usually represented by two variables related to the atomic position and one energetic variable, typically, the Gibbs free energy. The FEL maps were plotted using the *gmx sham* module, while the RMSD and RG were considered as the atomic position variables with respect to their average structure; figures were constructed using Wolfram Mathematica 12.1 [84]. The physicochemical, pharmacokinetic, and ADMET properties were calculated using the ADMETlab 2.0 server [85].

## 3. Results and Discussion

### 3.1. Molecular and ADMET Analyses of the Nicotine Analogs

An essential part of analyzing protein–drug interactions at the molecular level is that electrostatic forces are well represented. Much of the success of a drug in anchoring to the active site of the protein may depend on these types of interactions. As part of the methodology used in this work and to better depict protein–drug interactions, the five studied nicotine analog molecules (NAMs) were optimized using DFT calculations. Thus, the results made it possible to obtain the quantum atomic charges and incorporate them into the MD simulations in order to improve the protein–ligand interactions. Figure 1 shows the ESP surfaces obtained from both quantum and MD calculations. The names and the labels used to identify each NAM correspond to those used by Gandhi et al. in their research [44]. As can be seen, each molecule differs only in the chlorine atom positions in the phenyl ring structure. Thus, for better identification, each compound has been named according to the positions of these atoms: NIA (3, 5), NIB (2, 3), NIC (3, 4), NID (2, 5), and NIE (2, 4).

Analysis of quantum ESPs shows that all five NAMs have similar electrostatic behaviors. In general, these molecules have an electrophilic character at the ends of their structures with a central zone of high electron density. The high density occurs on the nitrogen of the pyridine ring and the oxygen molecules of the carbonyl groups. At the same time, the electropositive zones were found on the methyl group of the pyrrolic ring and the hydrogen atoms of the dichlorophenyl group. It is important to note that in the case of chlorine atoms, a slight electronegative character can be observed on their surface, which is increased or decreased depending on the position of these atoms. Thus, this electronegative character is increased for NIB and NIC compounds due to the continuous positions of the chlorine atoms on the ring.

The MM-ESP surfaces obtained by APBS calculations using the Hirshfeld atomic charges adequately represent the electrostatic character of the NAMs. However, in the area corresponding to the methyl group of the pyrrole ring, a slight increase in its electronegative character can be observed due to its proximity to the carbonyl oxygen. These results are key in the subsequent analyses, since they ensure a better representation of electrostatic protein–ligand interactions in calculations where it is not possible to deal with reactive systems. This methodology has been used in various works by our research group and also other groups, with excellent findings [86,87,88,89,90].

In order to assess the pharmacokinetics and toxicity of NAMs, their ADMET (absorption, distribution, metabolism, excretion, and toxicity) properties were evaluated using the ADMETlab 2.0 server [85]. Results are shown in Table 1.

Based on the drug-like soft rule, the physicochemical properties evaluation of the NAMs was positive. Among these properties, several are shared due to their intrinsic structure. For example, the molecular mass was 348.04 amu (optimal, 100∼600 amu). For hydrogen bonds, the number of acceptors was four (op., 0∼12), and there were no donors (op., 0∼7). The number of rotatable bonds was three (op., 0∼11), while there were 19 rigid bonds (op., 0∼30). NAMs had three rings in their structure (op., 0∼6), with a maximum number of six atoms in the biggest rings (op., 0∼18). Furthermore, NAMs only had six heteroatoms (op., 1∼15), which were electrically neutral (op., −4∼4). Moreover, they only had one stereocenter, and their polar surface area was 50.27 (op., 1∼140). In addition, the five compounds were found to be soluble in water (logS between −4∼0.5 log mol/L). Despite the logP values exceeding the limit value (0∼3), the values are within the permissible values at physiological pH (1∼3).

According to their medicinal chemical properties, NAMs are in the range of not-so-attractive molecules to design (low QED score). However, they are easily designable molecules (high SAScore), novel (MCE-18), with good absorption or permeability (according to the Lipinski rule), and good ADMET profiles (GSK rule and Golden Triangle). In addition, the five NAMs obtained excellent evaluations in absorption properties: high permeability in Caco-2 and MDCK cells; they are not P-glycoprotein (Pgp) inhibitors, nor are they Pgp substrates; and high absorption in the human intestine.

### 3.2. New MD Simulations Show High Structural Stability of the RvPs

There is evidence of the interaction between the Rv1417 and Rv2617c proteins (RvPs) with the Erp protein, one of the main virulence factors of Mtb. Klepp et al. consider that these proteins can form complex transient structures, and that their possible disruption could be used against *TB* [33]. Studies of the sequences of the RvPs place them as essentially membrane proteins [91,92]. Furthermore, our previous study showed that heterodimeric complexes are energetically more favorable under membrane conditions than in environments in which RvPs are not anchored [34].

Keeping in mind these proteins as targets, new MD simulations of the Rv1417, Rv2617c, and Erp systems were performed to obtain the structures and sites with the highest probability of interaction. The results confirm the high structural stability of the RvPs and the tendency of the Erp proteins to form globular structures in polar environments, as was described in our previous work (Table 2).

**Figure 2:**
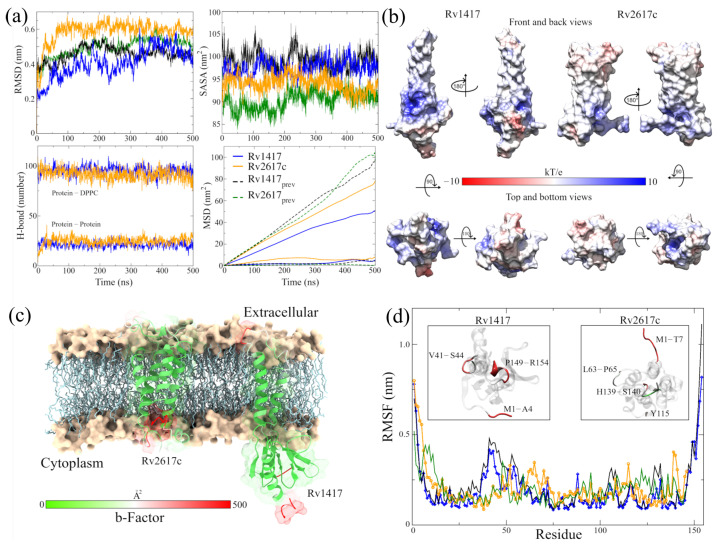
Analysis of the RvP structures in the new MD simulations. (**a**) Indicators of structural stability. The four graphs compare the structures of this work (blue and orange lines) with those presented previously (black and green lines). (**b**) Surfaces of the ESP of the final RvP structures. The range of colors corresponds to that described above. (**c**) B-factor values mapped onto protein structures. The red color indicates high fluctuations, the white color represents average values, and the green color represents minor fluctuations. (**d**) High-fluctuation residues identified on the RvP structures.

Although greater fluctuation was observed in the RMSD graph for the new structures, the values of the RMSF, the radius of gyration, and H bonds were very similar in both RvPs (Figure 2a and Table 2). The most remarkable difference was found in the formation of H bonds in Rv2617c, decreasing the number of intramolecular H bonds, compared to our previous results, going from 100 to 91 H bonds on average. However, there was a greater interaction with the membrane molecules (from 20 to 25 H bonds), which could explain the 6.7% increase in the radius of gyration about the *z*-axis.

An essential aspect of elucidating the interaction sites is the exposed area of the proteins. For this reason, solvent accessible surface area (SASA, Figure 2a) calculations were performed. The results show a slight increase in the exposed area outside the membrane for the Rv2617c protein compared to our previous measures (94.03 ± 1.88 by 90.36 ± 2.14 nm${}^{2}$). This increase is important for subsequent analyses of interaction sites. On the other hand, a decrease in the area was observed for the Rv1417 protein (97.58 ± 1.85 by 99.08 ± 2.13 nm${}^{2}$) due to lower exposure in the extracellular region.

A possible reason for the high fluctuation of the RvP structures could be their displacement through the membrane. When performing mean square displacement (MSD) calculations, both proteins shifted an order of magnitude faster than in previous studies. The calculated coefficients were D${}_{Rv1417}$ = 3.92 × 10${}^{-8}$ and D${}_{Rv2617c}$ = 1.50 × 10${}^{-8}$ cm${}^{2}$/s compared to the earlier values of D = 7.86 × 10${}^{-9}$ and D = 5.62 × 10${}^{-9}$ cm${}^{2}$/s, respectively. Meanwhile, the values for the displacement coefficients of the membrane molecules remained practically constant: 4.35 × 10${}^{-7}$ and 3.71 × 10${}^{-7}$ cm${}^{2}$/s compared to 5.20 × 10${}^{-7}$ and 4.86 × 10${}^{-7}$ cm${}^{2}$/s of the previous work.

Before the MD simulations with the NAMs, the electrostatic surfaces and per-residue fluctuations of the RvPs were analyzed to find possible interaction sites. Both proteins exhibit extensive hydrophobic areas (white color), characteristic of membrane proteins regarding the electrostatic surfaces (Figure 2b). However, the Rv1417 protein shows two areas of electrophilic character (blue color) on the β-sheet 1 region (comprising residues V10-R13 and P73–V93) and the region between the β-strand V98–S102 and α-helix 3 (K130–Y147). In addition, the nucleophilic regions were found on the β-turn A115–D117 and the final residues C152–R154. All these regions are located in the cytoplasmic domain. On the other hand, the Rv2617 protein exhibits differentiated electrostatic properties both in its cytoplasmic domain, which is electrophilic and in its extracellular (nucleophilic) domain. Notably, both hydrophilic characters are intensified inside the α helices that make up its structure.

From the vibrational analysis (Figure 2c,d), it can be seen that the residues located at the ends of both structures are the ones that fluctuate with greater intensity. However, on the extracellular domain, residues v41–S44 (Rv1417), L63–P65, and Y115 (Rv2617c) also show high values in their fluctuation. These results show that the main sites susceptible to interaction are located in the cytoplasmic region of the Rv1417 protein and both outer regions of the Rv2617c protein.

### 3.3. Building the RvP–NAM Interacting Systems

**Figure 3:**
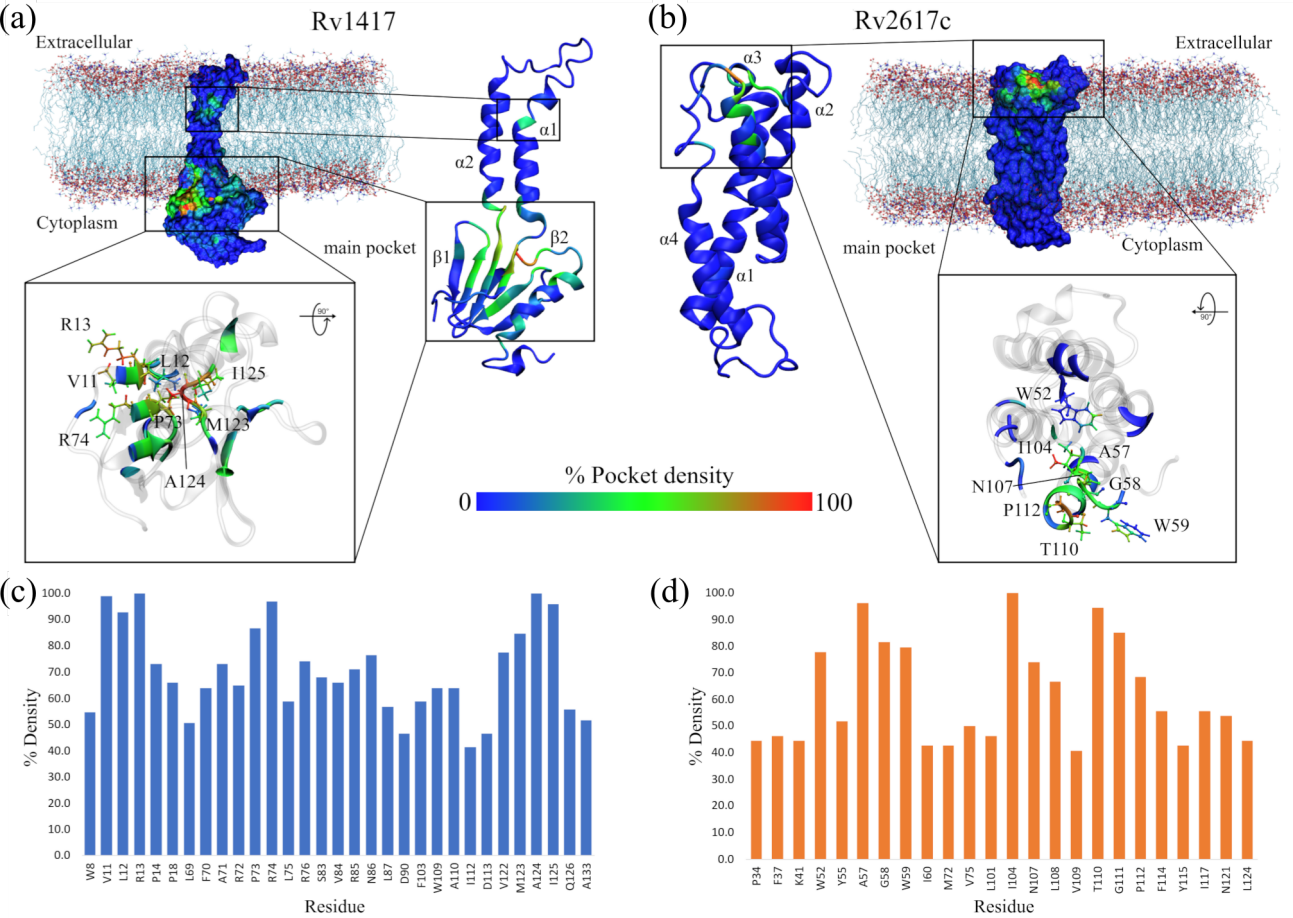
Highly conserved pockets throughout MD simulations. (**a**) Rv1417 structure. (**b**) RV2617 structure. The heat map is built based on the number of times each residue is part of a pocket for each analyzed frame. Thus, the blue color indicates a low density of forming a pocket, the green color indicates medium probability, and the red color indicates high probability. (**c**,**d**) Residues with the highest percentage of density calculated for the structures of the RvPs.

One of the main challenges in studying protein–drug interactions is finding sites with the highest binding probability. Unfortunately, experimentally, there are inherent limitations in studying active sites in membrane-associated proteins that make it difficult to locate these sites. Such is the case for the RvPs studied in this work, where information is scarce, making it difficult to find structural sites as starting points to analyze RvP–NAM interactions. In order to solve this issue, two different methodologies were followed to assess the affinity of the NAMs with the RvP structures. The first consisted of previously obtaining the interaction sites through pocket conservation analysis during the different MD trajectories (Figure 3). For this purpose, the MD trajectories were saved every ten nanoseconds and uploaded to the MDPocket server to obtain the hot spots of the protein structures. Then, local docking was performed using the pocket with the highest conservation probability, and the system with the best interaction energy was chosen for each RvP–NAM complex. The second methodology consisted of the solvation of each Rv system with 11 molecules of each NAM using the Gromacs *solvate* tool. Due to the fact that this tool randomly places the molecules inside the simulation box, care was taken that there were at least five molecules above and below the membrane in all initial configurations. Finally, all the complexes obtained by both methodologies were subjected to new 100 ns MD simulations.

#### 3.3.1. Highly Conserved Pockets during the MD Simulations of the RvP Systems

Figure 3a,b show, as heat maps, the probability that a residue in the RvPs is part of a pocket, which is preserved through the MD simulations. Based on this probability, possible active sites in proteins, including elusive binding sites or systems including membranes, can be elucidated [93,94,95]. The results show that for the Rv1417 protein, there are two differentiated pockets, a main one located in the cytoplasmic domain and a smaller one near the extracellular region. The first has a larger volume (2.56 nm${}^{3}$) and is located on the β1-sheet between residues V10–R13 and P73–V93. Furthermore, this pocket is located on the surface of the membrane, away from the region of greatest contact found in our previous work. (Figure 3a). The residues with the highest probability values (V11, L12, R13, P73, R74, W123, A124, and I125), i.e., with values greater than 80%, were located in this pocket (Figure 3c). The second pocket is located on the α1-helix, and is only made up of three residues, V30, V33, and L38. With a volume of 0.17 nm${}^{3}$, this pocket would be difficult for NAMs to access due to the volume of these molecules, which oscillates around 0.30 nm${}^{3}$.

On the other hand, for the Rv2617c structure, the main pocket is located in the extracellular region above the four α helices that make up the protein structure, with a volume of 0.94 nm${}^{3}$ (Figure 3b). The residues most likely to form the pocket are A57, G58, and W59, located between helices α1 and α2; I104 (α3-helix); and T110 and G111 (α4-helix, Figure 3d). Noteworthy is that, in this structure, residues A57, G111, P112, and F114 were also identified as part of the major contact sites in the Rv2617c–Erp heterodimers from our previous study.

These results reveal important information regarding the RvP structures. First, the Rv1417 protein is more susceptible to interactions in the cytoplasmic region under these simulation conditions, as its main pocket is located in this area. This putative active site is bulky and has an electrophilic character. However, its location makes it inaccessible to large structures, such as peptides or other proteins. However, recent studies on membrane-associated proteins have addressed the importance of occluded, cryptic, and hidden pockets as new therapeutic targets in drug design [93,95,96].

Second, in the case of the pocket found in the Rv2617c protein structure, it can be seen that it is located at a possible channel formed by the four α helices of the protein in the extracellular region. This result suggests that Rv2617c could have membrane transport protein functions, which would contribute to the understanding of the biological functions of this protein. In both cases, further exhaustive studies are recommended to validate these hypotheses.

#### 3.3.2. Stability Descriptors Show a High Structural Affinity in the RvP–NAM Complexes

For a better understanding of the following analyses, the complexes formed using the active sites will be named ASC14–NIx and ASC26–NIx, where the number indicates the protein and the letter x means the analogous nicotine molecule. The complexes formed by the solvation of NAMs will be named Sol14-NIx and Sol26–NIx, respectively. All RvP–NAM molecular complexes were subjected to MD simulations for 100 ns in NPT assemblies at 309.65 K and 1 bar pressure.

Table 3 and the graphs in Supplementary Figures S2 and S3 show the results of the stability descriptors of the simulations carried out. As expected, the systems have high structural stability, which is observed in the low fluctuation of the standard deviation values. In addition, the decrease in the RMSD values indicates that the final structures of the complexes do not differ much from the structures of minimum energy obtained in the protein–membrane simulations. However, it can be seen that the Sol26–NID complex exhibits the highest destabilization of the systems with an RMSD value of 0.37 ± 0.09 nm. This destabilization occurs between residues M1–F43 and Y115–P146, which comprise helices a1 and a4. The mean fluctuation of this complex was 0.19 ± 0.14 nm, the highest of all systems. It is important to highlight that in this complex, the lowest interaction was observed by H bonds with the NAMs of all the solvated complexes (0.01 ± 0.06 H bonds) and, on the contrary, the highest average number of H bonds with the membrane (19.27 ± 3.44). As for the other systems, the RMSF values show a decrease in the vibrations of the residues, indicating a possible stabilizing effect due to the presence of the NAMs. However, statistically, these values do not reflect this additional stability per se due to the high standard deviation.

On the other side, no significant changes in secondary structures or unfoldings that could cause protein denaturation was observed during molecular simulations (Table S1). In this sense, the values of both the radius of gyration and the intramolecular hydrogen bonds remained close to the values obtained in the simulations of the RvPs without the presence of the NAMs. Even for the complexes formed with the Rv2617c protein, intramolecular H bonds increased by more than 20% (ASC26–NIE). At the same time, although a decrease in the average electrostatic interactions between RvPs and membrane molecules was observed, these were small, being more evident in Sol26–NIx systems.

### 3.4. Assessment of the RvP–NAM Interactions

#### 3.4.1. Contact Analysis between the RvPs Residues and the NAMs

In a first evaluation to understand the nature of the interactions between RvPs and drugs, the simulation trajectories were analyzed to identify those residues in contact with each NAM. In this sense, H bonds and their percentage occupancy were determined using the last 50 ns of each simulation. In the case of the hydrophobic interactions, these were determined by analyzing the minimum energy structures of each molecular complex. In addition, calculations of the RvP–NAM interaction radius were performed to assess the solvating capacity of the drug molecules (protein+lig column in Table 3). The results are shown in Table 4.

At the same time, when analyzing the MD trajectories, it was observed that the drug molecule moved away from the active site in the ASC14–NIA, ASC26–NIB, and ASC26–NID systems. In the case of the ASC14–NIA system, the NIA drug remained on the protein by interacting with residues L19 and R74, and towards the end of the simulation, it moved away. However, for the ASC26–NIB and ASC26–NID systems, both NAMs left the site around 55 ns of simulation and no longer interacted with the protein. Therefore, in the subsequent analyses, these complexes were excluded from them.

Contact analysis showed that several residues are involved in the retention of the NAMs within the active sites of RvPs. In particular, for the systems with the Rv1417 protein, the arginines R13, R72, R74, and R85 played a determining role in this retention through H-bond-type interactions. Moreover, the calculations showed that the occupancy of the H bonds formed by R74 reached values close to 20%. Furthermore, in these complexes, the electrostatic interactions were significantly higher than those observed in the active site of Rv2617c, which were only observed with residues Q50 and N107 (NIA and NIC systems, respectively).

On the other hand, significant findings were found in systems solvated with NAM. Initially, one of the reasons for studying these systems was to verify the feasibility of NAMs interacting with found active sites without the need to place them directly on said sites. Under this scoop, trajectory analysis showed that the drug molecules interacted with the protein at various sites and remained close to the proteins by measuring the radius of gyration of RvP–NAM complexes (Table 3). The mean maximum protein–NAM radius was 3.14 ± 19 nm in simulation boxes with dimensions 111.32 × 11.36 × 11.23 nm on the *x*-, *y*-, and *z*-axis, respectively, and interactions were registered in all of them. However, the drug molecules did not interact with the proposed active site. In fact, only residues M123 and I125 with high pocket density interacted with the NIC drug. These results, together with the fact that this site is located in a region that is not easy to access, would suggest that it could be an elusive binding site requiring extensive movements to be exposed [93].

The results could also suggest a second hypothesis: this pocket could be an allosteric site. This assumption is proposed because the region of greatest interaction was between residues M1–D7, G78–S83, I92–S96, G106–W109, and L143–R154, located in the cytoplasmic region. All of these residues are found outside and on the periphery of the active site, so any interaction within this site could affect its function. This could also be a reference since, among the results of our previous work [34], this region has the highest probability of forming heterodimers with the Erp protein. Therefore, the disruption of the Rv1417–Erp interactions could occur in two ways, both in the periphery with the NAMs, and by affecting the proposed active site.

Moreover, the results showed that the NAMs also interacted with the Rv1417 protein in the extracellular region. Specifically, in Sol14–NIA Sol14–NIC complexes, the drugs mainly interacted with residues H32, Q49, Q53, and V54. Furthermore, interactions were observed in residues close to the secondary pocket of this protein (A31, H32, G35, and L37).

Regarding the systems formed with the Rv2617c protein, the analyses confirm that the region of greatest interaction is found in the extracellular region on the proposed active site. In addition, the interactions with the NAMs are distributed among the four main helices of the protein. Among the residues, Q50, H51, N53, M54, A57, N107, and P112 stand out because they provide H bond interactions, in addition to the fact that they were present in almost all the systems, including those that were built from the active site. In these systems, significant interactions were also observed on residues M1–P9 and residues H139, I143, and R145, all located in the cytoplasmic region.

#### 3.4.2. Energy and Electrostatic Analyses Show the Affinity of NAMs with the Active Sites

In order to assess the energetic affinity of NAMs with RvP structures, MMPBSA calculations were performed using the MD trajectories. The trajectories were saved every 100 ps, and the last 50 ns of each simulation were analyzed. In addition, these analyses were complemented by studying the vibrational energies per residue through the b-factor and obtaining the electrostatic maps of each complex. Finally, for their discussion, the results were divided into the systems built from the active sites and those solvated with NAMs.

The calculation of the BFEs of the RvPs complexes is shown in Table 5. The results show that all the complexes were energetically favorable, even in the systems where the drug moved away from the active sites (ASC26-NIB = −15.29 kJ /mol and ASC26-NID = −0.28 kJ/mol). For the ASC-14NIx complexes, the drugs NIB, NID, and NIE are the ones that showed a greater energetic affinity when reaching energies lower than −125 kJ/mol. In these three systems, the energy contribution of the electrostatic interactions was significant in drug retention since it exceeded some values obtained in the solvated systems (>41 kJ/mol). These results show that the active sites are energetically favorable to the interactions with the NAMs, being able to compete with the interactions on the protein surface.

**Figure 4:**
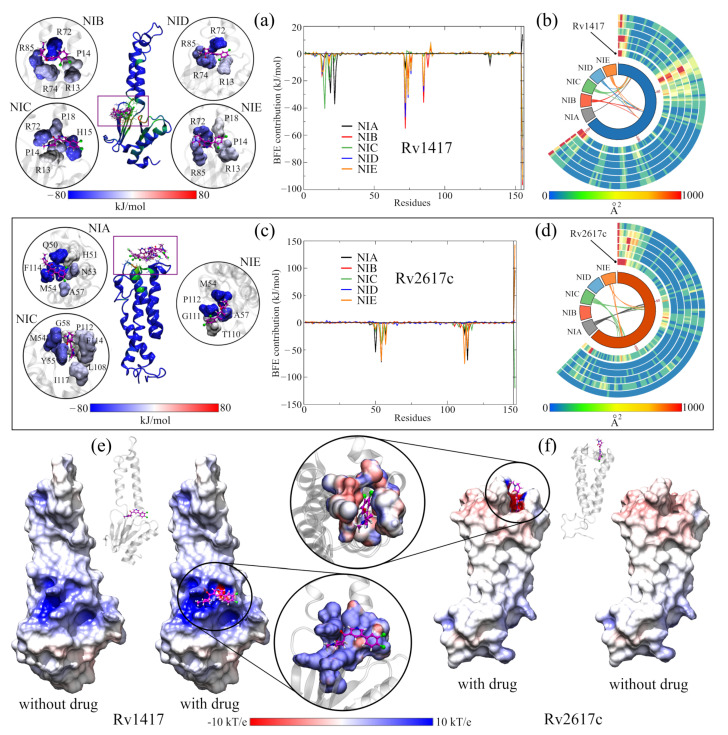
Analysis of RvP–NAM interactions. (**a**,**b**) Calculation of the free binding energies for the Rv1417 and Rv2617c systems. In both systems, the purple boxes indicate the pockets where the NAMs were docked. The circles show the interaction sites of each NAM and the residues with which they have contact. The color of the surfaces corresponds to the contribution to the interaction energy of each residue: blue for favorable energies and red for unfavorable energies. The graphs represent the energy analysis per residue. The colors of the lines were only used to differentiate each NAM. (**c**,**d**) Analysis of the b-factor of the proteins interacting with the NAMs. (**e**,**f**) Effect of NAMs on the ESP of RvPs. On the left, the protein is shown without the drug, and on the right, with the drug. The color scale used is red for electronegative regions and blue for electropositive regions. The white color indicates hydrophobic areas. In both representations, the RvP–NIC system is used.

Figure 4a shows the site where the local docking was carried out with the best-evaluated structures of this analysis. In the zoomed figures, the residues in direct contact with the NAMs are observed and colored according to the value of their BFE. In this analysis, the role of arginines R72, R74, and R85 in the interaction energies can be verified. In particular, R72 contributes favorable energies to the complexes, ranging from 10 kJ/mol (complex with NIA) to 55 kJ/mol (complex with NIB). Furthermore, in the graph of the contribution to the BFE per residue, four additional minima of favorable energies are observed, corresponding to residues H15 (−40.48 kJ/mol, NIC complex), L19 and Y22 (−29.09 and −37.23 kJ/mol, complex NIA). The other two minima correspond to arginines R74 and R85, whose values were −39.99 and −32.44 kJ/mol, respectively. The separation of the NIA drug from the active site can be seen in the black peak with positive energy at the end of the graph. Supplementary Table S2 shows the energy values of the ten residues with the highest contribution to BFE of all complexes, and the BFE surfaces are showed in Supplementary Figures S4 and S5.

In the case of ASC26–NIx complexes, the drug with the best interaction energy was NIC, which was almost 93% higher than the ASC26–NIA system, whose energy was −79.59 kJ/mol. Furthermore, of all the active site systems, this complex had the highest BFE in the MMPBSA calculations with only 5% electrostatic energy (−7.75 kJ/mol). Together with the contact analysis results, these findings suggest that the dominant interactions at this site are the hydrophobic character. Furthermore, the residues with the more significant contributions to the BFE had hydrophobic interactions: M54 and F114 for the ASC26–NIA complex (−72.55 and 69.09 kJ/mol, respectively) and M54 and P112 for the ASC26–NIE complex. (−75.89 and −70.19 kJ/mol). In the graph of the contributions to the BFE, the other two energetic minima correspond to residues Q50 (−54.41 kJ/mol) and A57 (40.57 kJ/mol) of the complexes with the drugs NIA and NIE, respectively.

As mentioned above, the vibrational movement of residues can be related to the ability of a protein to interact with other structures. Therefore, what was sought in the B-factor analyses, was a stabilizing effect of the NAMs toward the higher vibration residues in the RvPS. The results are shown in Figure 4b,d using Circos-type heat maps. In both cases, the results were positive, as a decrease in the B-factor values was observed. For protein Rv1417, in the initial and final residues, while in Rv2617c, this decrease was over residues P49–T67 and T110–Y115. For reference, the first heat map (the inner circle) shows the vibration of the residues in the simulations of the RvPs without the presence of NAMs.

Since previous analyses showed that at active sites, the electrostatic character plays a vital role in the interactions and retention of NAMs, the ESP surfaces of all complexes were calculated. In order to be able to compare the electrostatic effect of NAMs on protein surfaces, all ESPs were calculated without and with the presence of molecule drugs. The results shown in Figure 4e,f correspond only to the complexes formed with the NIC drug. The other figures can be reviewed in Supplementary Figures S6 and S7.

While slight changes can be seen on the ESP surfaces, these changes are primarily local. In ASC14–NIx complexes, the surfaces change mainly due to the presence of nitrogen, oxygen, and chlorine atoms, where the attraction is stronger given the electrophilic nature of the pocket. In contrast, in ASC26 systems, the local effect is more evident in the interactions with the NAMs. In both systems, the presence of the drug seems to reinforce the electrostatic character of the sites since, in the areas where there is no direct contact with the NAMs, the potential values decrease (ASC14–NIx) or approach zero (ASC26–NIx).

#### 3.4.3. Solvated Systems with NAMs

Following the same methodology to analyze the active site systems, the results in the solvated systems show significant energetic effects due to the free diffusion of the NAMs in the systems. First, the BFE calculations favored the RvP–NAM interaction (Table 5). The lowest energy was recorded for the Sol26–NID system (−103.82 kJ/mol), and the highest for the Sol14–NID complex (−312.57 kJ/mol). These results are significant since they could explain the influence that the position of the chlorine atoms in the phenyl ring would have. As mentioned above, chlorines have a slight electronegative character that, depending on their position, is reinforced when they are close together or distributed symmetrically when they are in opposite positions. In the case of the NID drug, the position is diametrically opposite, allowing the molecule to have two charged sites at this functional group. This charge distribution would allow a better electrostatic interaction on the protein surface, and could increase the affinity for hydrophilic sites. This distribution is also observed in the systems solvated with the drug NIA, where the chlorines are in positions 3 and 5 of the phenyl ring. In these systems, the second least favorable energy was recorded for the Sol26 complexes (−142.22 kJ/mol), and the second most favorable for the Sol14 complexes (−295.57 kJ/mol).

**Figure 5:**
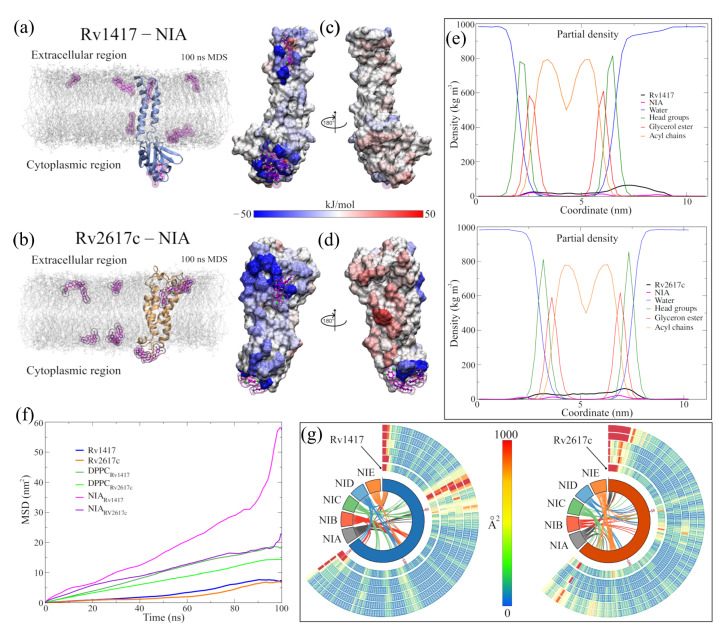
Analysis of the systems solvated by NAMs. (**a**,**b**) Final structures of the Sol14–NIA and Sol26–NIA systems. Proteins are shown in blue (Rv1417) and orange (Rv2617c) colors. NIA molecules are shown on magenta-colored surfaces; the lipid membrane is represented in transparent gray. (**c**,**d**) BFE values per residue mapped onto protein surfaces. Blue areas indicate favorable binding energies, and red areas represent repulsion. White areas indicate non-interacting residues. (**e**) Density profiles of different groups of the systems solvated by NIA. (**f**) Calculation of the diffusion coefficients of systems. (**g**) Heat maps as Circos plots of the b-factor of the different systems. Circles indicate (from the center out) protein structures and NAMs, RvPs without drugs, and systems NIA, NIB, NIC, NID, and NIE. The blue color indicates low vibration of the residue, while the red indicates high vibration. The central lines indicate the interactions between the protein residues and the different NAMs.

**Figure 6:**
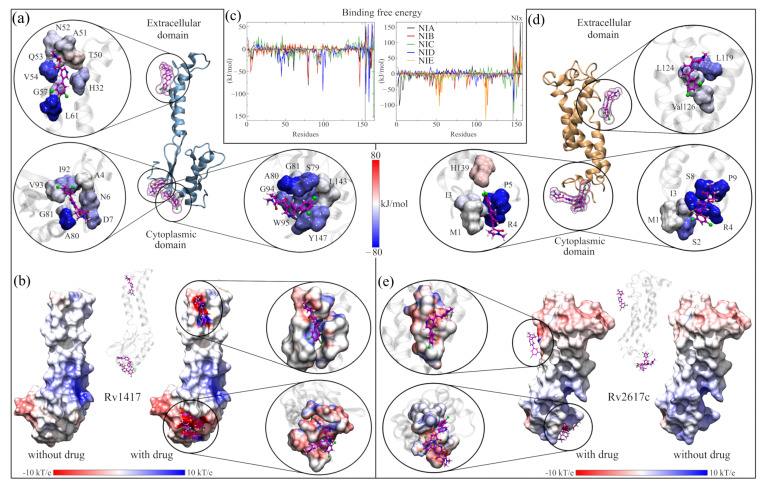
Analysis of systems solvated by NAMs. (**a**,**c**) BFE analysis of RvP–NAM binding sites. (**b**,**d**) Both subfigures show the complex formed with the NIA drug. (**e**) Surfaces of the ESP of the RvP structures with and without drug. Enlarged figures show binding sites. The same color codes and ranges for measurable properties used in the analyses of the complexes at the active sites are used in all figures.

Another point to highlight in the BFE analysis of the solvated systems is to observe that several residues interact with the drug molecules. That is, the contribution to the protein–drug binding energy is not only local but is distributed over the surface with which the drugs interact. To exemplify these observations, Figure 5 and Figure 6 show the analysis of the systems obtained using the NIA molecule as the solvent drug. Figure 5a,b show the final structures of the simulations, including the RvP unions with the NIA molecules (colored in magenta). In both systems, the extracellular region presents an internalization of the NIA molecules, while in the cytoplasmic region, the interactions occur on the surface. In addition, it can be observed that the drug interacts with the membrane molecules, and some even begin to penetrate the lipid layers. Due to drug diffusion, the protein surfaces show the binding sites with the protein and those areas with which it interacted attractively (blue spots) and repulsively (red spots) (Figure 5c,d). Furthermore, in all solvated systems, it was observed that when a drug interacted laterally with the protein, the residues on the other side repelled the interactions with the other molecules. These surfaces can be consulted in Supplementary Figures S8 and S9.

To ensure that the behavior of membrane molecules did not affect the results due to possible unpacking or phase changes, density, diffusion, and area per lipid calculations were performed (Table 6). The results show great stability of the lipid molecules and that there are no structure losses. The difference in the densities of the head groups and the glycerol esters is mainly due to the number of membrane α helices of each protein. However, in all systems, the density values remain constant. Furthermore, diffusion values are within the expected values for membranes that do not have cholesterol molecules in their structure, whose order of magnitude is close to 1 × 10${}^{-7}$ cm${}^{2}$/s [97,98]. Finally, for the area per residue, the values are close to the experimental values for mammalian lipid membranes, which exhibit values around 42 Å${}^{2}$ [99]. Figure 5e,f show the density profiles and diffusion values of the Sol14–NIA and Sol26–NIA systems, which illustrate the aforementioned results. Noteworthy is that the diffusion coefficient values of the NAMs in the systems were also calculated, producing values that oscillate by 2.28 × 10${}^{-7}$ cm${}^{2}$/s and 1.40 × 10${}^{-6}$ cm${}^{2}$/s. These values, although relatively low, are very close to the diffusion coefficients of drugs with similar molecular masses (×10${}^{-6}$ cm${}^{2}$/s) [100,101].

Figure 5g shows the effect of NAMs on the vibrational energies of RvPs. Taking the heat map of the protein without drugs as a reference, it can be observed that in the Rv1417 protein, the vibration only decreases in the last residues and increases in the extracellular zone. This is because drug molecules in this region tend to interact with the flanking regions rather than the exposed residues. For the Rv2617c protein, a decrease in the fluctuations of the systems solvated by the NIA, NIB, and NIC drugs is observed. However, systems with NID and NIE increased their vibrations in the cytoplasmic region because, in these systems, there were no interactions with NAMs. These results demonstrate that the presence of NAMs has a stabilizing effect when interacting with the RvP structures.

The local effect of the NAMs with the interacting residues can be observed by analyzing the contributions per residue to the BFE and the electrostatic surfaces. Figure 6a,b exemplify the impact of the NIA drug on the Rv1417 protein. In this system, three NIA molecules were observed to interact with the protein, one in the extracellular region and the other two in the cytoplasmic region. Analysis of the BFE per residue shows that all three NIA molecules possess an affinity to neighboring residues. However, at the junction of the extracellular region, there is a certain repulsion due to the T50 residue (10.11 kJ/mol) that can destabilize the complex. On the other hand, the residues with the highest binding energy are found in the cytoplasmic region. Therefore, the presence of the two NIA molecules leads to an increased affinity in that region. The electrostatic map also shows important alterations in the contact zones (Figure 6b). In both sites, an increase in electronegativity is observed, which is more evident in the cytoplasmic region due to the two NIA molecules. These changes are essential since the affinity of Rv1417 changes from hydrophobic structures to systems with an electrophilic character. The energy distribution shows considerable interactions in the systems, both attractive and repulsive (Figure 6c). In the Sol14–NIx systems, it was observed that the system with the NID drug has the residue with the most significant energetic contribution to the BFE of this complex, I99 (−93.68 kJ/mol). However, the residues that interacted in at least four systems were P5, A80, I92, and G94, all in the cytoplasmic region.

On the other hand, in the Sol26–NIA system, although three NIA interaction sites were also observed with the Rv2617c protein, they are energetically less intense than those of the Rv1417 protein. As in the Sol14–NIA system, there was one interaction in the extracellular region and two in the cytoplasmic region. Located in the lateral area of the protein, extracellular binding is only favored by three residues: L119, L124, and V126 (−29.48, −12.70, and −10.55 kJ/mol, respectively). Instead, the interactions in the cytoplasmic region are favored by residues with high values of BFE: R4, P5, S8, and P9 (−102.01, −79.58, −51.77, and 47.90 kJ/mol, respectively). Only the H130 residues had repulsive energies in this region (14.16 kJ/mol). The electrostatic effect of this drug is similar to that of the Sol14–NIA system; contact causes the area to take on an electronegative character instead of its hydrophobic character observed in the simulations without the presence of the drug. In addition, for the Sol26NIx systems, the highest favorable energies were reached with residues P112 and M54 (−147.88 and −103.23 kJ/mol) with the drug NIE, R4 (−102.01 kJ/mol) with NIA, and F114 (−100.43 kJ/mol) with NIB (Supplementary Table S2). Furthermore, residues M54 and Q50 presented interactions in three systems.

A particular case is Sol26–NIE, in which eight NIE molecules were observed in interaction with the Rv1617c protein. Six molecules interacted in the extracellular region and two in the cytoplasmic region, although laterally. In this complex, the electrostatic effect of the area with the most significant contact is notorious since it changes the electrostatic properties of the protein, making it highly electronegative. In addition, due to the number of NIE molecules, this site was the one that generated the most attractive interaction.

### 3.5. Energy Analysis of RvP–Erp Heterodimers in the Presence of NAMs

**Figure 7:**
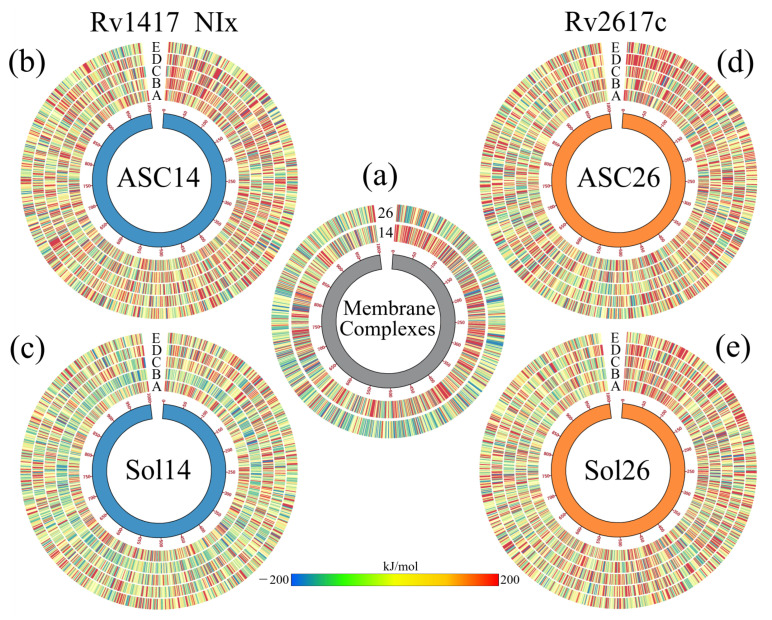
Comparison of the interaction energies of the best 1000 RvP–Erp dimeric complexes. The energies were obtained from molecular docking calculations. (**a**) Energies of the dimers obtained in our previous analyses. Numbers 14 and 26 indicate the protein used as the receptor, Rv1417 or Rv2617c, respectively. (**b**,**c**) Energy for the Rv1417–Erp dimers. (**d**,**e**) Energy for the Rv2617c–Erp dimers. All graphs were built on the same scale of energy values.

In order to assess the results obtained, the final structures of all the systems were evaluated through molecular docking calculations. For these couplings, the protein–NAM–membrane systems were taken as the receptor, and the Erp minimum energy structure as the ligand. The results are shown in Figure 7. To obtain the heat maps, the energies calculated in the refinement process with FireDock were taken into account, allowing the flexibility of the coupled structures. For better sampling, 50 Monte Carlo cycles and a scaling radius of 0.8 were used for minimization, optimization, and scoring [74,75]. In all Circos heat maps, the bars colored in blue indicate favorable binding energy, and the red color indicates unfavorable.

Our previous work showed that the RvP–Erp dimers have better interaction energies when the RvPs are anchored in the membrane [34]. Furthermore, the Rv2617c–Erp complexes are energetically more favorable than those formed by the Rv1417 protein (Figure 7a). Taking these results as a reference, our calculations show that the presence of the NAMs also influenced the coupling energies. In general, the dimers with the best interaction were those formed with Rv1417 as a receptor, both in the active site and solvated systems (Figure 7b,c). However, when comparing their energies with those of reference, it can be seen that there was a greater number of dimers with favorable energies, especially for the system with NIB. This result seems contradictory since the NIE molecules interacted on the previously observed high-contact regions for this system. Furthermore, the systems in which the NAMs interacted at the active site seem to affect the interaction energies. This opens up the possibility that allosteric effects may exist at this site that structurally affect the protein, and should be addressed in greater detail in subsequent work.

On the other hand, remarkable effects were observed in systems with the Rv2617c protein as the receptor. In this sense, Figure 7d,e show a decrease in dimers with favorable energies. This may be explained by the fact that the active site of Rv2617c is exposed in both systems, which makes interaction with NAMs more likely, thus affecting dimer formation. Although the energy values obtained in these molecular couplings must be refined with more in-depth studies, they can be an excellent basis to take into account for the use or design of drugs.

## 4. Conclusions

There is evidence that the Erp protein, one of the virulence factors of the *Mycobacterium tuberculosis* bacterium, interacts with the Rv1417 and Rv2617c proteins to form various molecular complexes. One of the theories suggests that the disruption in forming these complexes could help treat TB. In the present work, we show the results of our search for interaction sites in the Rv1417 and Rv2617c proteins that could be potential therapeutic targets. In addition, we have used five nicotine analog molecules (NAMs) to determine whether they can be used to neutralize these sites and prevent the interaction of these proteins with the Erp protein.

The analyses reveal that there are two pockets with the potential to be active sites, one located in the cytoplasmic region of the Rv1417 protein, and the second in the extracellular region of Rv2617c. The first one could be an allosteric or elusive binding site, since an excellent relative affinity with the drugs used was observed. A decrease in dimers with favorable energies could lead in this direction. Furthermore, under solvation conditions, no drug interacted with residues at this site. In contrast, the active site of Rv2617 is confirmed to be occupied by NAMs in both simulation conditions. The low number of Rv2617c dimers with favorable energies suggests that it could be a good candidate as a therapeutic target.

More in-depth calculations and experimental tests are necessary to support the findings presented herein. However, we believe that the methodology can be used as a tool in searching for active sites in systems where there is limited experimental information.

## Figures and Tables

**Table 1:** ADMET prediction of the five nicotine analogs obtained using the ADMETlab 2.0 server.

Property	Model Name	Predicted Value				
		NIA	NIB	NIC	NID	NIE
Physicochemical	logS	−4.014	−3.912	−4.062	−3.871	−3.923
	logP	3.492	3.254	3.418	3.271	3.307
	logD	2.481	2.191	2.405	2.257	2.334
Medicinal	QED	0.627	0.627	0.627	0.627	0.627
Chemistry	SAScore	2.996	2.995	2.906	2.954	2.945
	NPScore	−0.866	−0.911	−0.962	−0.941	−0.979
Absorption	Caco-2 Permeability	−4.656	−4.595	−4.631	−4.622	−4.620
	MDCK Permeability	1.3 × 10${}^{-5}$	2.9 × 10${}^{-5}$	2.4 × 10${}^{-5}$	2.0 × 10${}^{-5}$	2.0 × 10${}^{-5}$
	Pgp inhibitor	- - -	- - -	- - -	- -	- -
	Pgp substrate	- - -	- - -	- - -	- - -	- - -
	HIA	- - -	- - -	- - -	- - -	- - -
Distribution	PPB	0.950	0.916	0.921	0.916	0.918
	VD	1.14 3	1.280	1.181	1.275	1.226
	Fu	0.263	0.587	0.489	0.544	0.523
Metabolism	CYP1A2 inhibitor	+++	+++	+++	+++	+++
	CYP1A2 substrate	++	++	++	++	++
	CYP2C19 inhibitor	++	+++	+++	+++	+++
	CYP2C19 substrate	+	-	-	-	-
	CYP2C9 inhibitor	+	++	+	+	+
	CYP2C9 substrate	- -	+	-	-	-
	CYP2D6 inhibitor	-	-	-	+	-
	CYP2D6 substrate	+	+	++	++	+
	CYP3A4 inhibitor	+++	+++	+++	+++	+++
	CYP3A4 substrate	+	+	+	+	+
Excretion	CL	3.150	3.568	3.325	2.804	2.846
	t${}_{1/2}$	0.260	0.215	0.176	0.186	0.171
Toxicity	hERG Blockers	+	+	++	+	++
	H-HT	-	-	-	-	-
	DILI	+	++	++	++	++
	AMES Tox.	- - -	- - -	- - -	- - -	- - -
	Rat Oral Acute Tox.	- -	-	-	- -	- -
	FDAMDD	-	-	-	-	-
	Skin Sensitization	- -	- -	- -	- -	- -
	Carcinogenicity	- - -	- -	- -	- -	- -
	Eye Corrosion	- - -	- - -	- - -	- - -	- - -
	Eye Irritation	- - -	- - -	- - -	- - -	- - -
	Respiratory Tox.	- - -	- -	- -	- - -	- - -

For the classification endpoints, the prediction probability values are transformed into six symbols: 0–0.1 (- - -), 0.1–0.3 (- -), 0.3–0.5 (-), 0.5–0.7 (+), 0.7–0.9 (++), and 0.9–1.0 (+++).

**Table 2:** Comparison of the stability descriptors of the RvPs and Erp proteins obtained in the present work and those obtained in the previous work.

System	^a^ RMSD	^a^ RMSF	^a^ RG				H Bonds	H Bonds + DPPC
			Total	*x*-axis	*y*-axis	*z*-axis		
**Membrane and Water—500 ns**								
Rv1417	0.41 ± 0.08	0.19 ± 0.12	2.13 ± 0.02	2.00 ± 0.04	1.99 ± 0.03	1.03 ± 0.05	96 ± 5	21 ± 3
${}^{b}$ *Prev.*	*0.46 ± 0.05*	*0.22 ± 0.14*	*2.14 ± 0.02*	*2.03 ± 0.03*	*2.01 ± 0.03*	*1.00 ± 0.05*	*93 ± 5*	*21 ± 4*
Rv2617c	0.59 ± 0.08	0.20 ± 0.11	1.85 ± 0.03	1.70 ± 0.03	1.68 ± 0.03	1.05 ± 0.04	91 ± 6	25 ± 4
*${}^{b}$ Prev.*	*0.50 ± 0.06*	*0.20 ± 0.07*	*1.85 ± 0.03*	*1.73 ± 0.04*	*1.71 ± 0.03*	*0.98 ± 0.04*	*100 ± 5*	*20 ± 3*
Erp	0.70 ± 0.10	0.34 ± 0.17	2.01 ± 0.03	1.66 ± 0.15	1.58 ± 0.15	1.66 ± 0.15	110 ± 8	-
*${}^{b}$ Prev.*	*0.65 ± 0.12*	*0.33 ± 0.17*	*2.04 ± 0.02*	*1.72 ± 0.12*	*1.55 ± 0.15*	*1.70 ± 0.14*	*114 ± 8*	-

^a^ Values in nanometers. All values were obtained from the last 300 ns of the MD simulations. ^b^ The stability values of prior work are shown in italics.

**Table 3:** Stability Descriptors of the *Mtb* proteins and system complexes.

System	RMSD ^a^	RMSF ^a^	RG ^a^		H Bonds		
			Protein	Protein + lig	Intra	Inter–lig	Inter–DPPC
ASC14–NIx							
NIA	0.22 ± 0.03	0.15 ± 0.08	2.12 ± 0.02	2.11 ± 0.02	95 ± 5	0.13 ± 0.36	19.74 ± 2.94
NIB	0.23 ± 0.04	0.15 ± 0.09	2.14 ± 0.02	2.12 ± 0.02	94 ± 5	0.62 ± 0.58	19.54 ± 2.91
NIC	0.23 ± 0.04	0.16 ± 0.08	2.13 ± 0.02	2.12 ± 0.02	94 ± 5	0.43 ± 0.51	20.92 ± 2.98
NID	0.19 ± 0.03	0.14 ± 0.06	2.13 ± 0.02	2.12 ± 0.02	93 ± 4	0.39 ± 0.54	21.85 ± 2.91
NIE	0.22 ± 0.04	0.15 ± 0.06	2.12 ± 0.02	2.10 ± 0.02	93 ± 4	0.19 ± 0.42	21.87 ± 3.04
ASC26–NIx							
NIA	0.30 ± 0.04	0.15 ± 0.06	1.89 ± 0.02	1.91 ± 0.02	107 ± 4	0.13 ± 0.42	19.27 ± 3.21
NIB	0.22 ± 0.03	0.13 ± 0.06	1.83 ± 0.02	1.86 ± 0.04	105 ± 5	0.12 ± 0.33	19.75 ± 2.96
NIC	0.28 ± 0.04	0.17 ± 0.07	1.90 ± 0.02	1.90 ± 0.02	100 ± 6	0.03 ± 0.16	19.27 ± 3.18
NID	0.23 ± 0.04	0.16 ± 0.06	1.83 ± 0.03	1.88 ± 0.04	104 ± 5	0.00 ± 0.00	20.17 ± 3.03
NIE	0.21 ± 0.02	0.11 ± 0.05	1.83 ± 0.02	1.85 ± 0.02	109 ± 5	0.00 ± 0.00	19.84 ± 3.20
Sol14-NIx							
NIA	0.27 ± 0.05	0.17 ± 0.11	2.13 ± 0.02	2.92 ± 0.19	92 ± 4	0.49 ± 0.71	21.73 ± 3.74
NIB	0.27 ± 0.04	0.15 ± 0.08	2.13 ± 0.02	2.93 ± 0.10	94 ± 5	1.44 ± 0.92	19.96 ± 3.21
NIC	0.28 ± 0.05	0.16 ± 0.08	2.11 ± 0.02	3.14 ± 0.16	97 ± 5	0.40 ± 0.61	17.90 ± 3.05
NID	0.25 ± 0.04	0.15 ± 0.09	2.13 ± 0.02	2.88 ± 0.18	93 ± 5	1.00 ± 1.03	20.79 ± 3.46
NIE	0.26 ± 0.03	0.15 ± 0.08	2.14 ± 0.02	3.02 ± 0.26	94 ± 5	0.44 ± 0.56	20.17 ± 3.27
Sol26-NIx							
NIA	0.22 ± 0.04	0.14 ± 0.06	1.88 ± 0.02	2.75 ± 0.20	104 ± 5	0.38 ± 0.62	18.09 ± 2.58
NIB	0.25 ± 0.04	0.14 ± 0.07	1.88 ± 0.02	2.71 ± 0.12	102 ± 5	0.40 ± 0.60	17.78 ± 2.94
NIC	0.23 ± 0.04	0.14 ± 0.07	1.90 ± 0.02	2.68 ± 0.10	99 ± 5	0.87 ± 0.77	16.54 ± 2.69
NID	0.37 ± 0.09	0.19 ± 0.14	1.91 ± 0.03	3.14 ± 0.19	101 ± 6	0.01 ± 0.06	19.27 ± 3.44
NIE	0.19 ± 0.02	0.12 ± 0.06	1.82 ± 0.02	2.30 ± 0.13	103 ± 5	0.56 ± 0.71	18.20 ± 2.86

^a^ in nanometers. All values were obtained from the last 50 ns of the MD simulations.

**Table 4:** Contact analysis in the NAM–RvP complexes.

NAM	Active Site Complexes		Solvated Complexes	
	Rv1417	Rv2617c	Rv1417	Rv2617c
NIA	L19, **R74 (9.18)**	**Q50 (11.2)**, H51, N53, M54, A57, F114	A4, N6, **D7 (11.8)**, H32, T50, A51, D52, Q53, V54, G57, L61, S79, A80, G81, I92, V93, G94, **W95 (4.0)**, **S96 (22.9)**, L143, Y147	M1, **S2 (7.5)**, I3, **R4 (7.18)**, P5, **T7 (11.7)**, **S8 (5.7)**, P9, L119, L124, V126, H139
NIB	R13, P14, **R72 (12.2)**, **R74 (7.4)**, **R85 (16.4)**	-	T2, A3, P5, **N6 (56.9)**, **D7 (25.2)**, G78, S79, **A80 (6.7)**, G81, L82, S83, I92, V93, G94, W95, S96, E97, L143, R146, Y147, R148	F42, P49, Q50, **H51 (6.7)**, **N53 (6.3)**, M54, Y55, Q70, **Y73 (8.19)**, A77, P112, **F114 (9.9)**
NIC	R13, P14, H15, P18, **R72 (15.9)**	M54, Y55, G58, **N107 (25.9)**, L108, P112, F114, I117	L27, A31, H32, G35, L37, V46, V47, F48, **Q49 (7.0)**, Q53, V54, A55, **F103 (12.0)**, G106, **R108 (10.9)**, W109, M123, I125, A127, V128	**I3 (8.4)**, A19, L26, P49, **Q50 (27.6)**, M54, Y55, **N107 (10.8)**, L108, P112, F114, V126, G127, A130, R133, L134, **S140 (17.1)** I143, R145
NID	R13, R72, **R74 (19.9)**, R85	-	**W95 (12.0)**, **S96 (14.4)**, I99, G100, V101, S102, D150, L151, **A153 (10.6)**, **R154 (21.2)**	L36, L39, F43, L45, I60, N61, V64, A99, W100, A102, G103
NIE	R13, P14, P18, R72, **R74 (6.0)**, R85	M54, A57, T110, G111, P112	M1, **T2 (6.8)**, **A3 (6.6)**, A4, P5, **N6 (14.4)**, A80, G81, I92, V93, G94, S96	A10, **Q50 (15.3)**, **H51 (10.8)**, **N53 (15.0)**, L63, L108, V109, T110, G111, P112, G113, F114, Y115, I129, A132, R133

The bold letters indicate H bond interactions, and the numbers in parentheses are their percentage occupancy measured in the last 50 ns of the MD trajectories.

**Table 5:** Average MM/PBSA free energies of RvP–NAM interactions at 100 ns.

Complex	$\Delta E_{vW}$	$\Delta E_{Elec}$	$\Delta E_{PS}$	$\Delta E_{SASA}$	BFE
ASC14–NIx					
NIA	−52.78 ± 2.30	11.53 ± 1.68	3.51 ± 1.57	−7.28 ± 0.27	−45.01 ± 4.37
NIB	−100.81 ± 0.54	−67.36 ± 0.94	42.12 ± 0.55	−13.69 ± 0.05	−139.74 ± 1.23
NIC	−103.28 ± 0.64	−15.42 ± 1.71	42.70 ± 0.94	−13.27 ± 0.06	−89.27 ± 1.32
NID	−86.25 ± 0.84	−49.19 ± 1.94	17.17 ± 1.56	−12.15 ± 0.10	−130.42 ± 2.15
NIE	−100.00 ± 0.75	−41.62 ± 1.28	28.09 ± 1.04	−12.45 ± 0.07	−125.99 ± 1.64
ASC26–NIx					
NIA	−82.89 ± 0.85	−13.30 ± 1.22	27.38 ± 0.98	−10.79 ± 0.10	−79.59 ± 2.93
NIB	−12.50 ± 1.52	0.61 ± 0.55	−1.53 ± 1.22	−1.87 ± 0.21	−15.29 ± 3.69
NIC	−165.29 ± 0.83	−7.75 ± 0.87	40.14 ± 0.68	−15.34 ± 0.10	−148.24 ± 2.00
NID	−0.52 ± 0.08	−2.15 ± 0.27	2.55 ± 0.98	−0.16 ± 0.07	−0.28 ± 2.49
NIE	−76.85 ± 0.78	−3.93 ± 0.82	21.05 ± 0.70	−9.42 ± 0.10	−69.15 ± 2.84
Sol14–NIx					
NIA	−323.35 ± 2.26	−43.97 ± 1.55	107.35 ± 2.13	−32.59 ± 0.25	−292.57 ± 6.22
NIB	−281.80 ± 2.12	−70.73 ± 1.75	98.53 ± 1.77	−28.78 ± 0.24	−282.77 ± 5.74
NIC	−203.07 ± 4.48	−30.14 ± 2.13	46.94 ± 1.95	−26.86 ± 0.60	−213.13 ± 12.23
NID	−347.67 ± 3.79	−91.54 ± 3.83	167.41 ± 2.64	−40.78 ± 0.40	−312.57 ± 8.25
NIE	−138.95 ± 2.17	−20.37 ± 1.33	59.84 ± 1.86	−15.87 ± 0.27	−115.35 ± 6.74
Sol26–NIx					
NIA	−156.83 ± 2.90	−47.89 ± 2.63	83.49 ± 2.14	−21.00 ± 0.38	−142.22 ± 7.87
NIB	−313.60 ± 4.69	−74.58 ± 3.85	149.28 ± 2.98	−41.41 ± 0.53	−280.30 ± 11.13
NIC	−308.47 ± 2.46	−30.20 ± 2.07	87.67 ± 1.96	−32.08 ± 0.27	−283.09 ± 7.56
NID	−120.69 ± 2.32	−0.75 ± 0.90	32.08 ± 1.16	−14.46 ± 0.29	−103.82 ± 6.83
NIE	−290.07 ± 2.67	−32.44 ± 2.29	90.30 ± 2.41	−34.52 ± 0.31	−266.73 ± 6.98

All values are in kJ·mol^−1^.

**Table 6:** Different properties of lipids when interacting with the systems of interest.

Protein	NAM	$\rho_{\max}$			Diffusion			Area per Lipid	
		H-G	G-E	A-Ch	DPPC	Protein	NIx	Up	Down
Rv1417	NIA	800.15	596.65	791.40	4.95 ± 0.91	2.15 ± 2.28	6.15 ± 2.18	48.61 ± 0.49	49.37 ± 0.40
	NIB	788.40	588.95	789.35	5.27 ± 0.55	1.53 ± 0.42	6.30 ± 3.03	48.78 ± 0.48	49.56 ± 0.36
	NIC	791.10	590.20	786.95	5.24 ± 1.44	2.00 ± 0.44	7.11 ± 0.34	48.91 ± 0.68	49.64 ± 0.65
	NID	784.30	585.87	786.31	4.34 ± 0.49	0.20 ± 0.34	5.03 ± 2.50	48.87 ± 0.43	49.58 ± 0.42
	NIE	794.82	592.44	788.52	3.95 ± 0.71	2.01 ± 3.08	13.95 ± 1.7	48.76 ± 0.69	49.48 ± 0.79
Rv2617c	NIA	837.15	611.15	780.50	2.41 ± 0.28	1.56 ± 2.46	2.99 ± 0.03	48.91 ± 0.65	49.46 ± 0.56
	NIB	805.48	585.74	775.22	4.58 ± 0.33	1.96 ± 1.63	9.66 ± 10.08	48.96 ± 0.41	49.38 ± 0.60
	NIC	798.79	580.63	775.10	5.15 ± 2.07	0.21 ± 0.12	2.28 ± 0.39	48.34 ± 0.58	49.10 ± 0.66
	NID	822.28	599.22	779.33	4.48 ± 0.41	0.27 ± 0.14	5.54 ± 4.13	48.75 ± 0.48	49.00 ± 0.43
	NIE	817.93	594.18	778.69	4.12 ± 0.42	0.88 ± 0.09	2.16 ± 0.08	49.16 ± 0.32	49.35 ± 0.38

Density is given in kg/m${}^{3}$; diffusion × 10${}^{-7}$ cm^2^/s and area per lipid in Å^2^. $\rho_{\max}$ is the average maximum density measured in both lipid layers; H-G means head groups; G-E, glycerol ester; and A-Ch, acyl chains. Diffusion was measured in the *x*-*y* plane for the DPPC molecules and the protein. For drugs, the diffusion value corresponds to the three Cartesian coordinates. For the area per lipid, Up is the top lipid layer, and Down is the bottom lipid layer.

## Data Availability

Not applicable.

## Supplementary Materials

The following supporting information can be downloaded at: https://www.mdpi.com/article/10.3390/biom13020248/s1, Figure S1: Secondary structure of RvPs, α corresponds to alpha-helices and β to beta-sheets; Figure S2: Stability descriptors for the systems built with the drugs into the active sites. The colors used are to identify each NAM: NIA, black; NIB color red; NIC, green color; NID, blue color; and NIE, orange; Figure S3: Stability descriptors for the systems built with NAMs as a solvent. The colors used are to identify each NAM: NIA, black; NIB color red; NIC, green color; NID, blue color; and NIE, orange; Figure S4: Binding sites of NAMs with Rv1417 protein. The front view is shown on the right and a 180° turn on the left. Blue surface areas represent attractive interactions, while red surface areas represent repulsive interactions; Figure S5: Binding sites of NAMs with Rv2617c protein. The front view is shown on the right and a 180° turn on the left. Blue surface areas represent attractive interactions, while red surface areas represent repulsive interactions; Figure S6: Electrostatic potential effects of NAMs on the Rv1417 surfaces. On the left, the protein is presented without the drug, and on the right with it. Both were rotated 180º with respect to each other to better visualize the two faces of these proteins. The color scale used was red for electronegative regions and blue for electropositive regions. The white color indicates hydrophobic areas; Figure S7: Electrostatic potential effects of NAMs on the Rv2617c surfaces. On the left, the protein is presented without the drug, and on the right with it. Both were rotated 180º with respect to each other to better visualize the two faces of these proteins. The color scale used was red for electronegative regions and blue for electropositive regions. The white color indicates hydrophobic areas; Figure S8: Binding sites of NAMs to the Rv1417 protein in solvated systems. The front view is shown on the right and a 180° turn on the left. Blue surface areas represent attractive electrostatic interactions while the red surface areas represent repulsive electrostatic interactions; Figure S9: Binding sites of NAMs to the Rv2617 protein in solvated systems. The front view is shown on the right and a 180° turn on the left. Blue surface areas represent attractive electrostatic interactions while the red surface areas represent repulsive electrostatic interactions; Figure S10: Electrostatic potential effects of NAMs on the Rv1417c surfaces in a solvated system. On the left, the protein is presented without the drug, and on the right with it. Both were rotated 180º with respect to each other to better visualize the two faces of these proteins. The color scale used was red for electronegative regions and blue for electropositive regions. The white color indicates hydrophobic areas; Figure S11: Electrostatic potential effects of NAMs on the Rv2617c surfaces in a solvated system. On the left, the protein is presented without the drug, and on the right with it. Both were rotated 180º with respect to each other to better visualize the two faces of these proteins. The color scale used was red for electronegative regions and blue for electropositive regions. The white color indicates hydrophobic areas; Table S1: Secondary structures conservation of the Rv1417 and Rv2617c proteins in isolation and bounded with the NAMs; Table S2: Top 10 residues that contribute to the binding free energy in the RvPs–NAMs interaction.

## Abbreviations

The following abbreviations are used in this manuscript:

Mtb	*Mycobacterium tuberculosis*
TB	Tuberculosis
Erp	Exported repetitive protein
NAMs	Nicotine analog molecules
RvPs	Rv1417 and Rv2617c proteins
DFT	Density functional theory
MD	Molecular dynamics
FEL	Free energy landscape
DDPC	Dipalmitoylphosphatidylcholine
BFE	Binding free energies
ESP	Electrostatic potential
MM-ESP	Electrostatic potential surfaces within the molecular mechanics framework
MM/PBSA	Molecular mechanics Poisson–Boltzmann surface area
ADMET	Absorption, distribution, metabolism, excretion, and toxicity)
ASC14–NIx	Active site complex of NAMs with Rv1417
ASC26–NIx	Active site complex of NAMs with Rv2617c
