# Supplementary material for: Study of the Rv1417 and Rv2617c Membrane Proteins and Their Interactions with Nicotine Derivatives as Potential Inhibitors of Erp Virulence-Associated Factor in Mycobacterium tuberculosis: An In Silico Approach
Source: Biomolecules. 2023 Jan 28;13(2):248. doi: 10.3390/biom13020248 (PMC9953637; doi:10.3390/biom13020248)
Supplement: Supplementary file 1 [file biomolecules-13-00248-s001.zip › biomolecules-2075622-supplementary.pdf]

Study of the Rv1417, and Rv2617c Membrane Proteins and Their Interactions with Nicotine Derivatives as Potential Inhibitors of Erp Virulence-Associated Factor in Mycobacterium Tuberculosis: an in silico approach

January 28, 2023

Jorge Alberto Aguilar-Pineda[1], Camilo Febres-Molina[1,2], Cinthia C. Cordova-Barrios[3], Lizbeth M. Campos-Olazával[4], Bruno A. Del-Carpio-M[1], Flor Ayqui-Cueva[1], Pamela L. Gamero-Begazo [1,2], and Badhin Gómez[1,3]*.

[1] Centro de Investigación en Ingeniería Molecular— CIIM, Universidad Católica de Santa María, Urb. San José s/n—Umacollo, Arequipa, 04013, Peru.
[2] Doctorado en Fisicoquímica Molecular, Facultad de Ciencias Exactas, Universidad Andres Bello, Santiago de Chile, 8370134, Chile.
[3] Departamento de Ciencias Farmacéuticas, Bioquímicas y Biotecnológicas, Universidad Católica de Santa María, Urb. San José s/n, Umacollo, Arequipa 04013, Peru.
[4] Facultad de Arquitectura e Ingeniería Civil y del Ambiente, Universidad Católica de Santa María, Urb. San José s/n, Umacollo, Arequipa 04013, Peru.

Keywords: Alzheimer disease, Apolipoprotein E, amyloid-β, Enoxaparin, Molecular Dynamics.

*Author to whom correspondence should be addressed.
Electronic mail: bgomez@ucsm.edu.pe (B.G.); Tel.: +51-982895967

Supplementary Figures

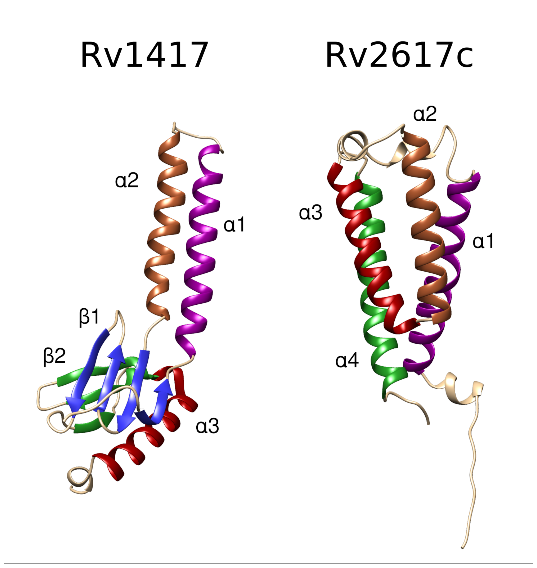

Figure S1. Secondary structure of RvPs, α corresponds to alpha-helices and β to beta-sheets.

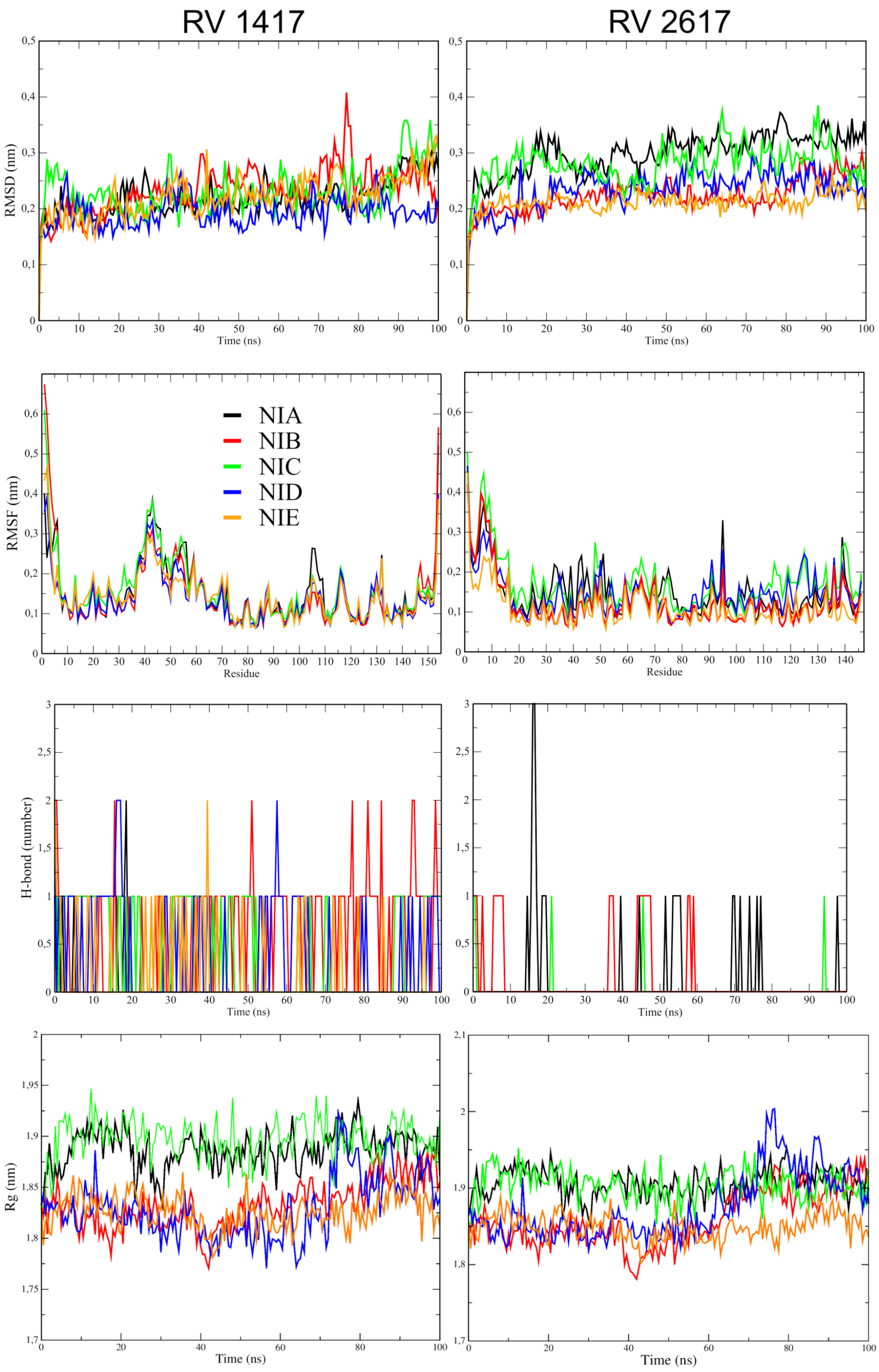

Figure S2. Stability descriptors for the systems built with the drugs into the active sites. The colors used are to identify each NAM: NIA, black; NIB color red; NIC, green color; NID, blue color; and NIE, orange.

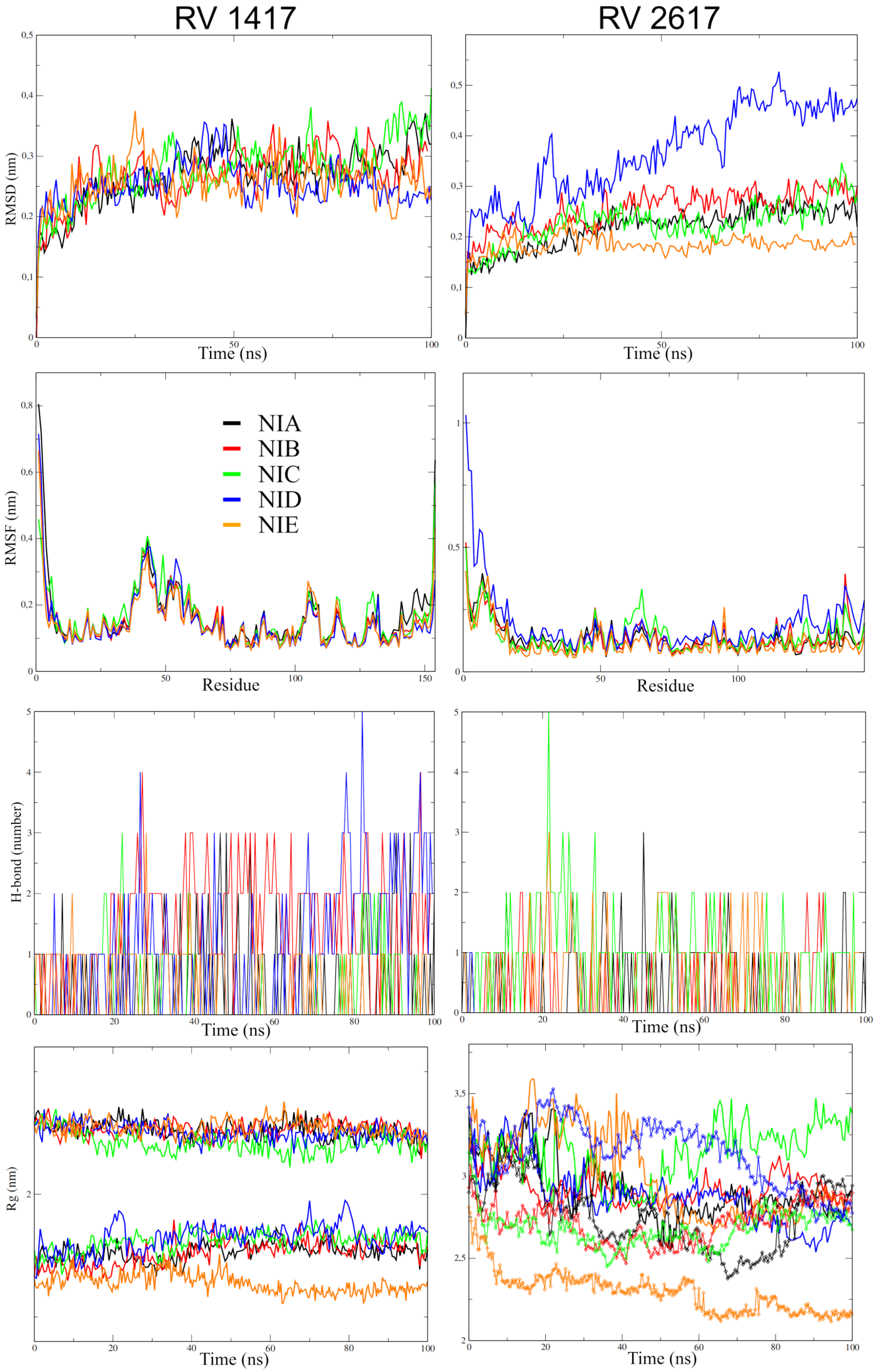

Figure S3. Stability descriptors for the systems built with NAMs as a solvent. The colors used are to identify each NAM: NIA, black; NIB color red; NIC, green color; NID, blue color; and NIE, orange.

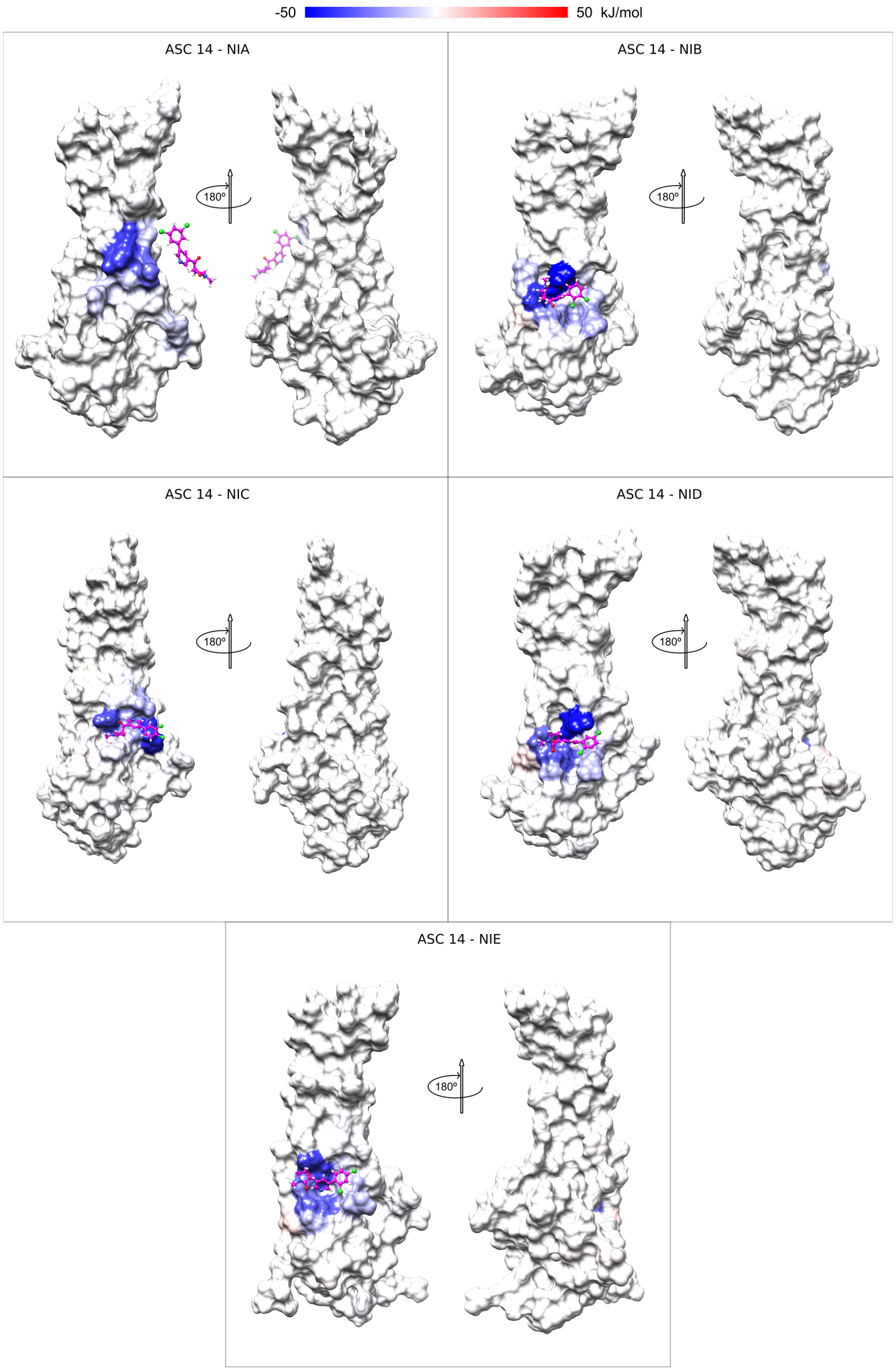

Figure S4. Binding sites of NAMs with Rv1417 protein. The front view is shown on the right and a 180° turn on the left. Blue surface areas represent attractive interactions, while red surface areas represent repulsive interactions.

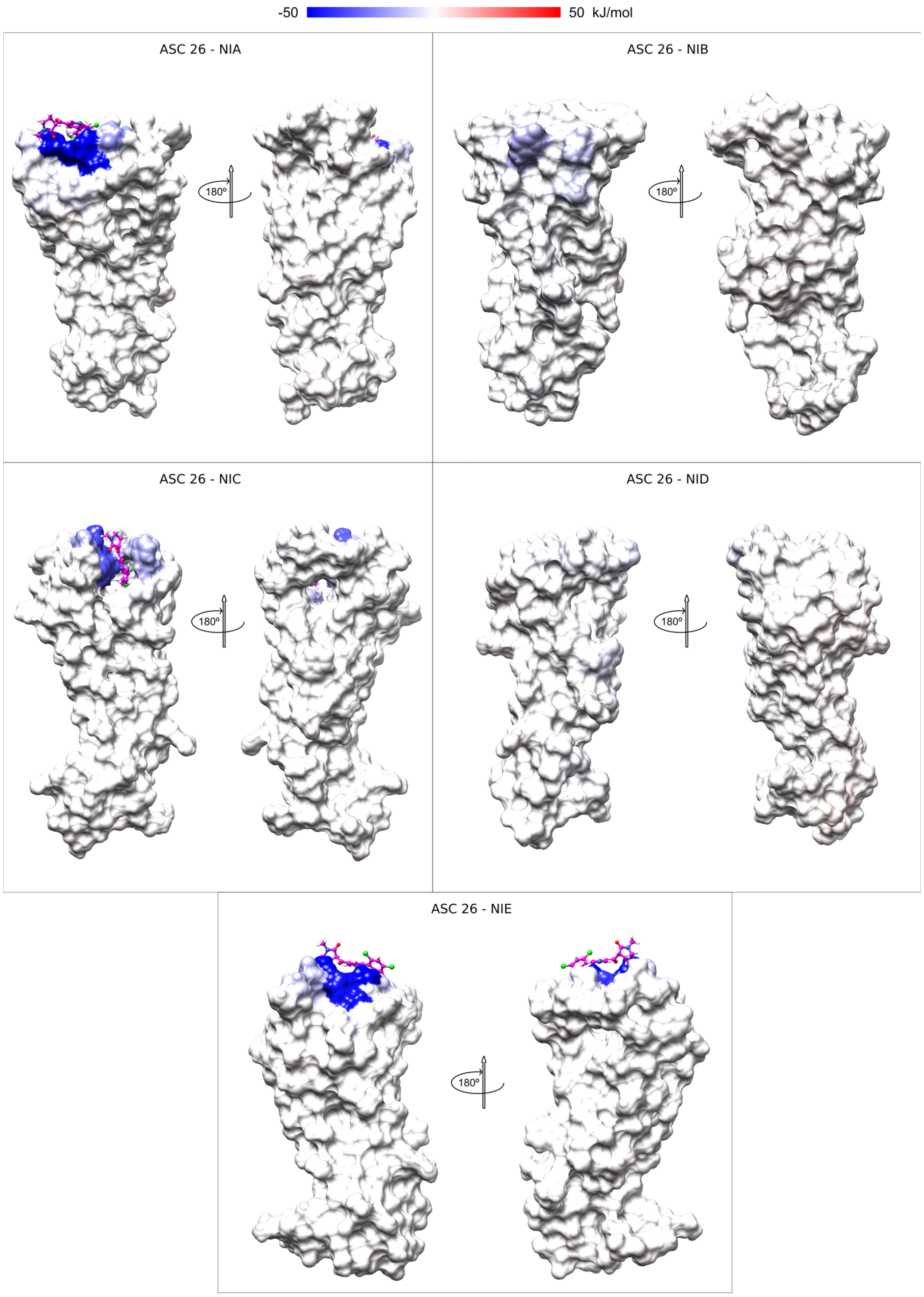

Figure S5. Binding sites of NAMs with Rv2617c protein. The front view is shown on the right and a 180° turn on the left. Blue surface areas represent attractive interactions, while red surface areas represent repulsive interactions.

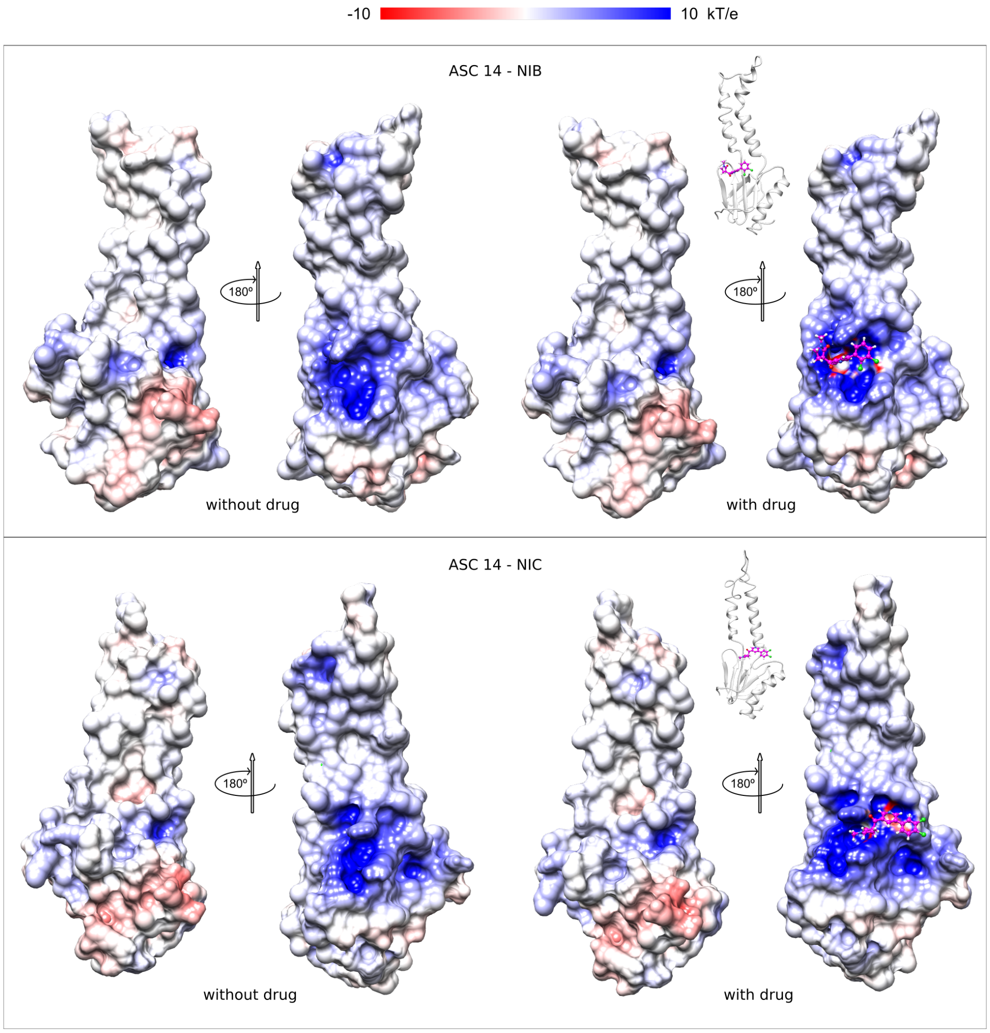

Figure S6-1. Continue next page.

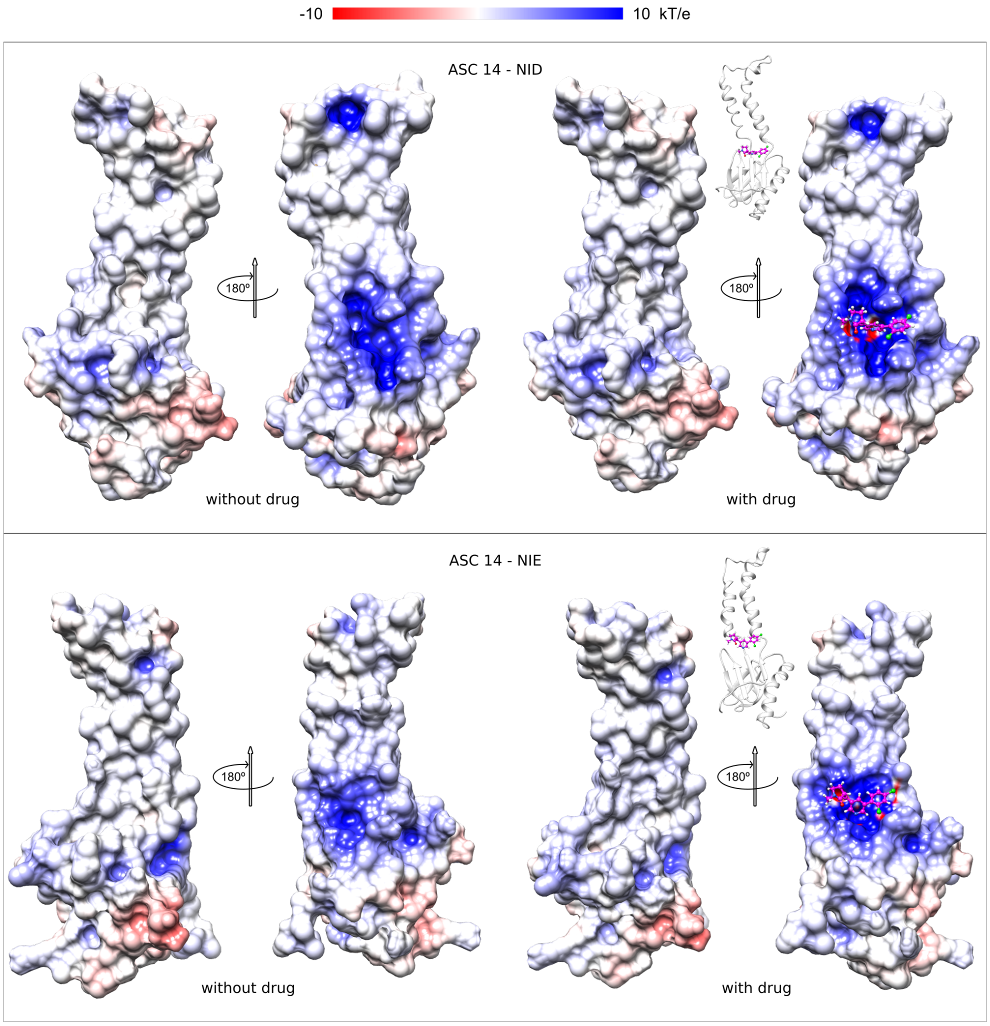

Figure S6-2. Electrostatic potential effects of NAMs on the Rv1417 surfaces. On the left, the protein is presented without the drug, and on the right with it. Both were rotated 180º with respect to each other to better visualize the two faces of these proteins. The color scale used was red for electronegative regions and blue for electropositive regions. The white color indicates hydrophobic areas.

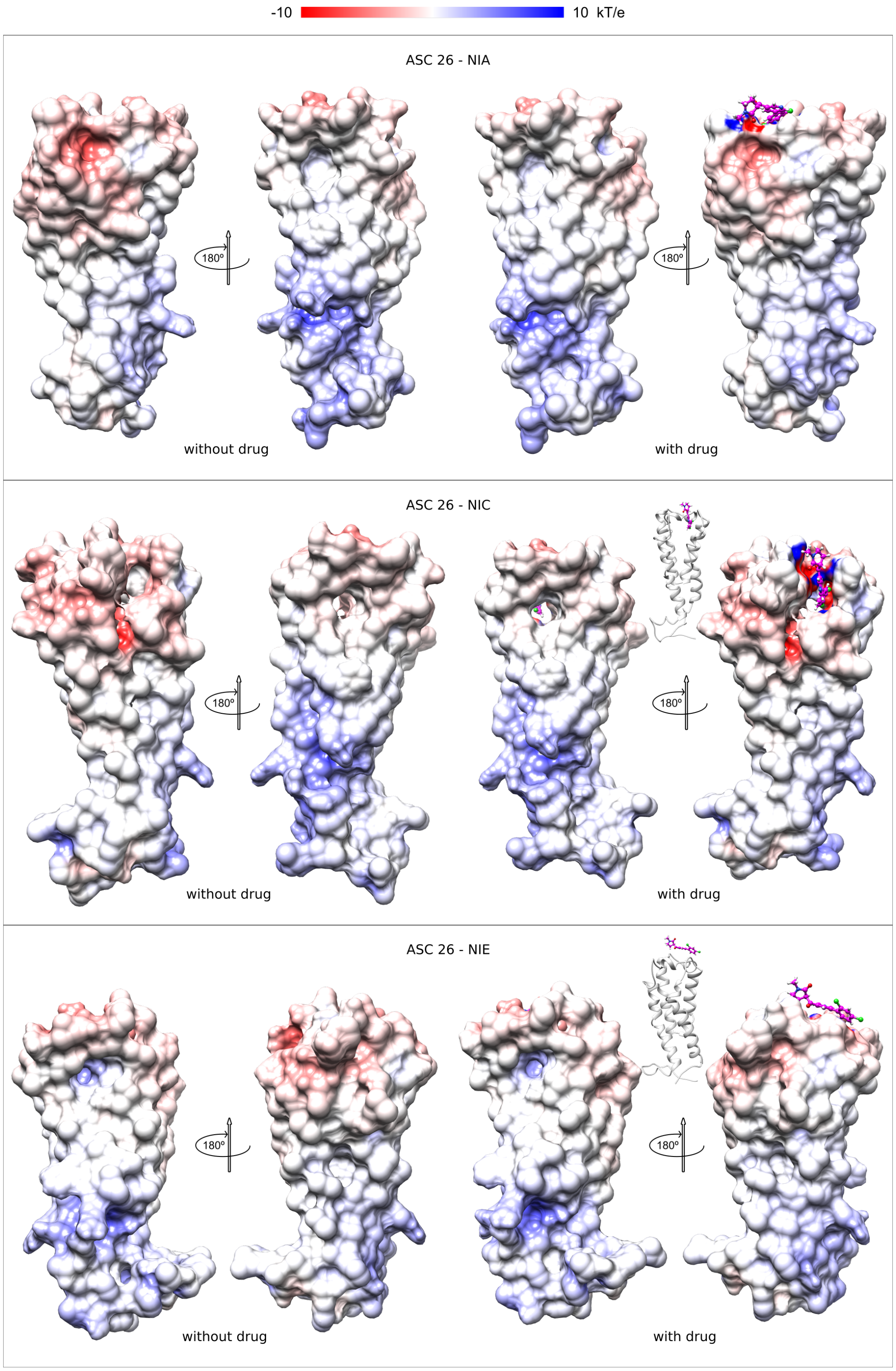

Figure S7. Electrostatic potential effects of NAMs on the Rv2617c surfaces. On the left, the protein is presented without the drug, and on the right with it. Both were rotated 180º with respect to each other to better visualize the two faces of these proteins. The color scale used was red for electronegative regions and blue for electropositive regions. The white color indicates hydrophobic areas.

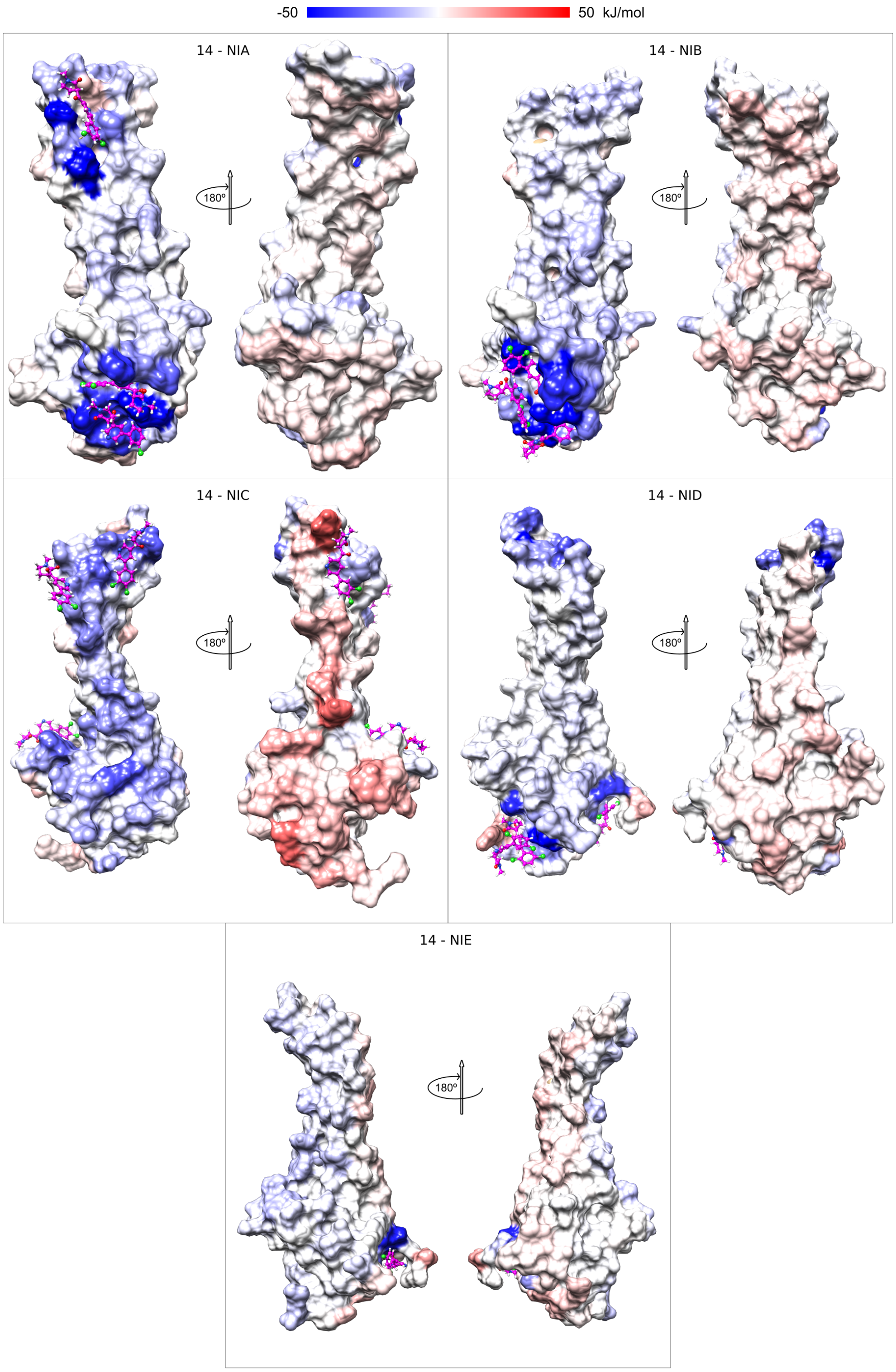

Figure S8. Binding sites of NAMs to the Rv1417 protein in solvated systems. The front view is shown on the right and a 180° turn on the left. Blue surface areas represent attractive electrostatic interactions while the red surface areas represent repulsive electrostatic interactions.

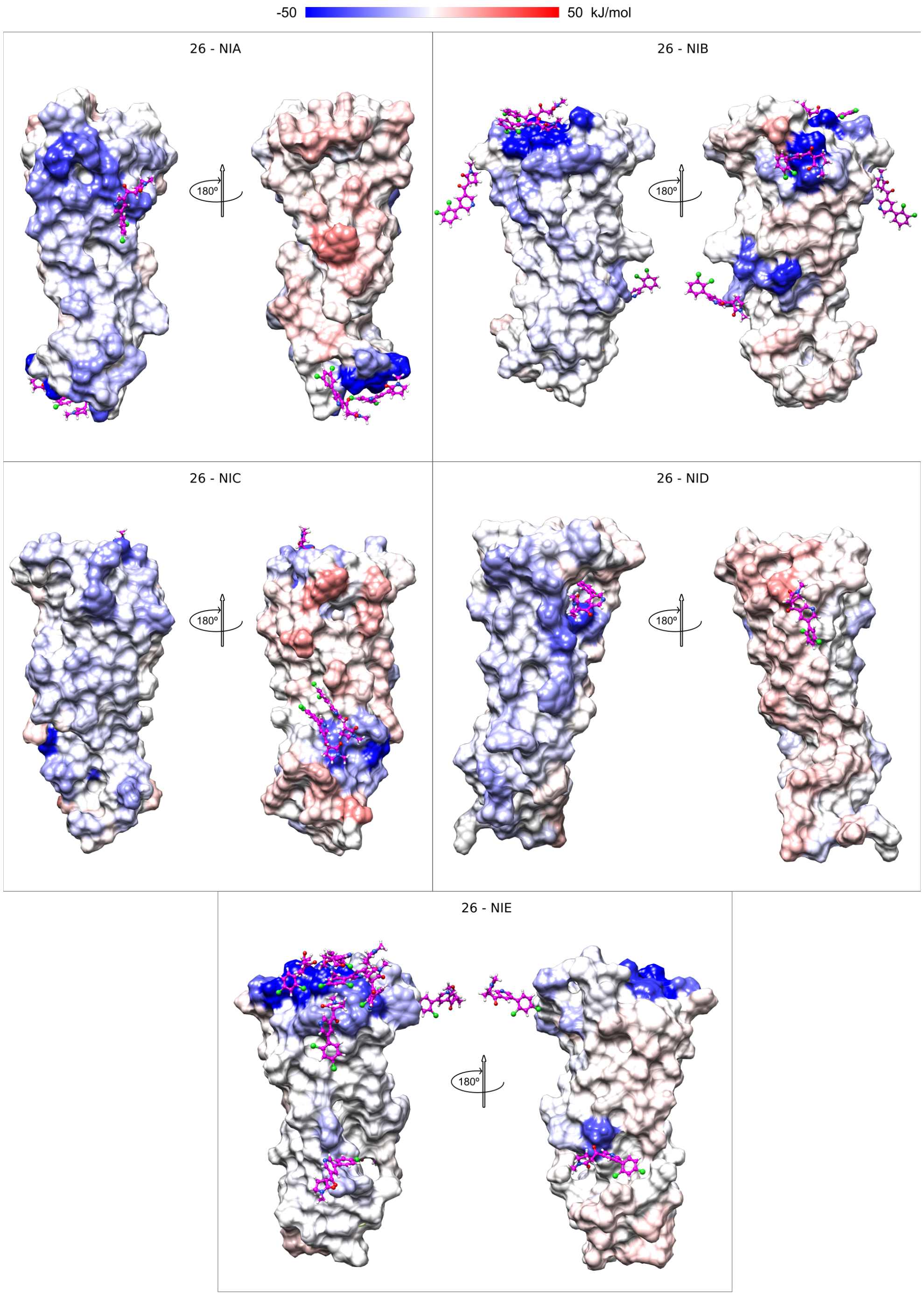

Figure S9. Binding sites of NAMs to the Rv2617 protein in solvated systems. The front view is shown on the right and a 180° turn on the left. Blue surface areas represent attractive electrostatic interactions while the red surface areas represent repulsive electrostatic interactions.

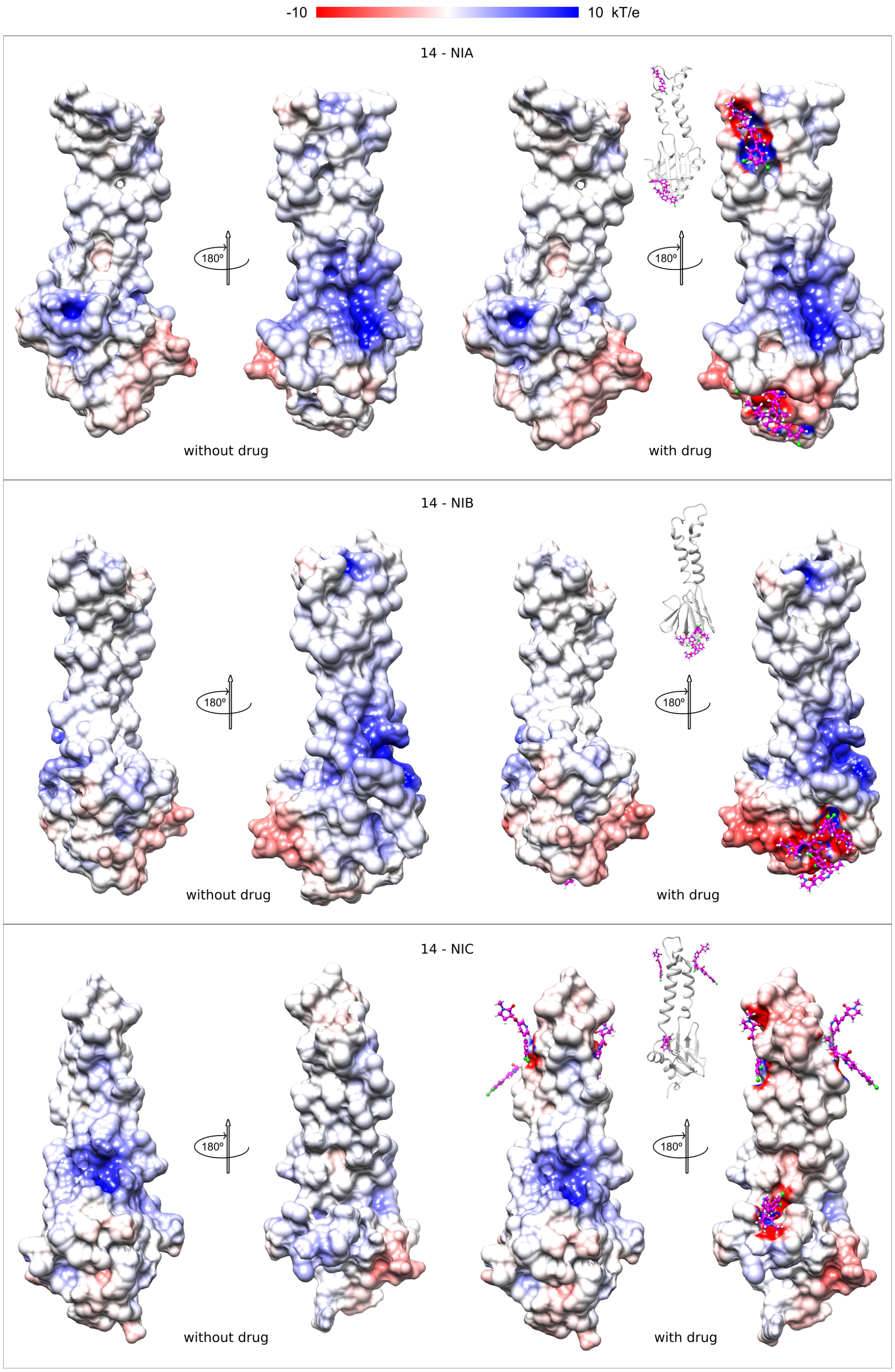

Figure S10-1. Continue next page.

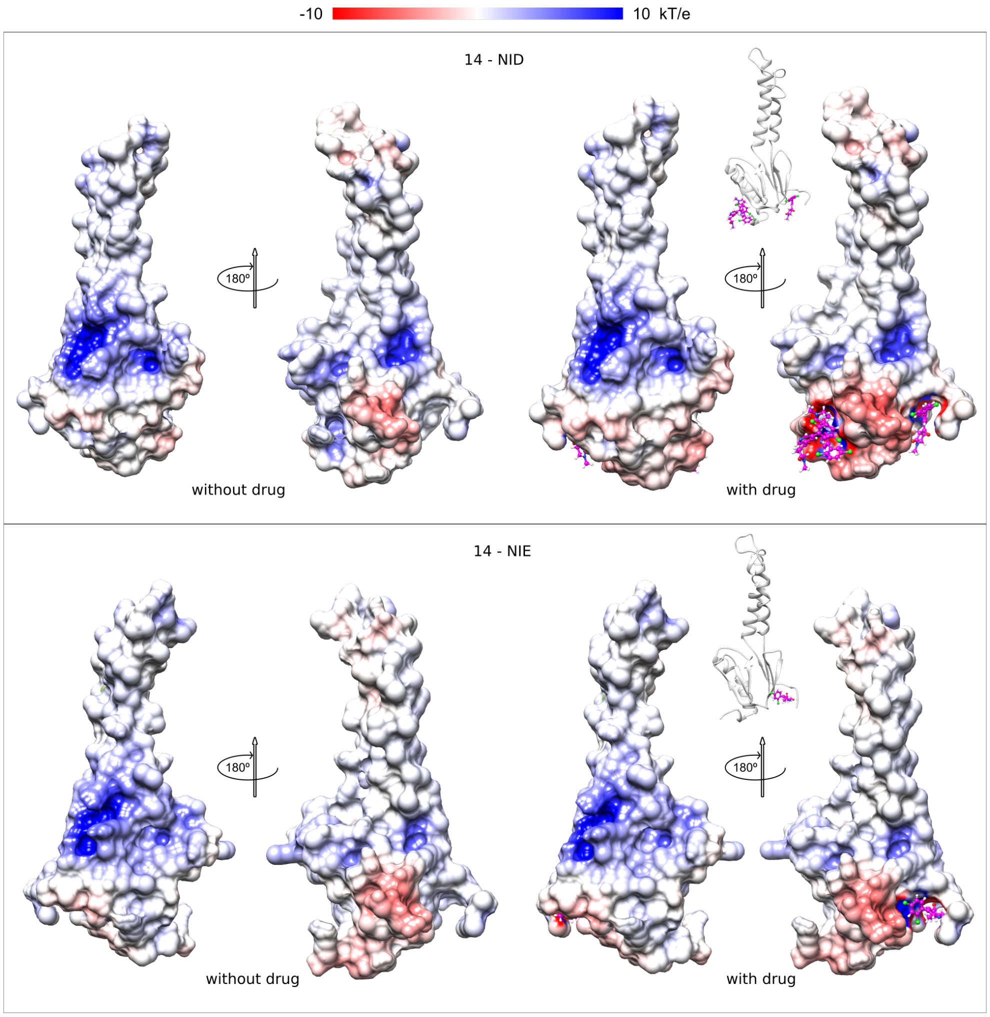

Figure S10-2. Electrostatic potential effects of NAMs on the Rv1417c surfaces in a solvated system. On the left, the protein is presented without the drug, and on the right with it. Both were rotated 180º with respect to each other to better visualize the two faces of these proteins. The color scale used was red for electronegative regions and blue for electropositive regions. The white color indicates hydrophobic areas.

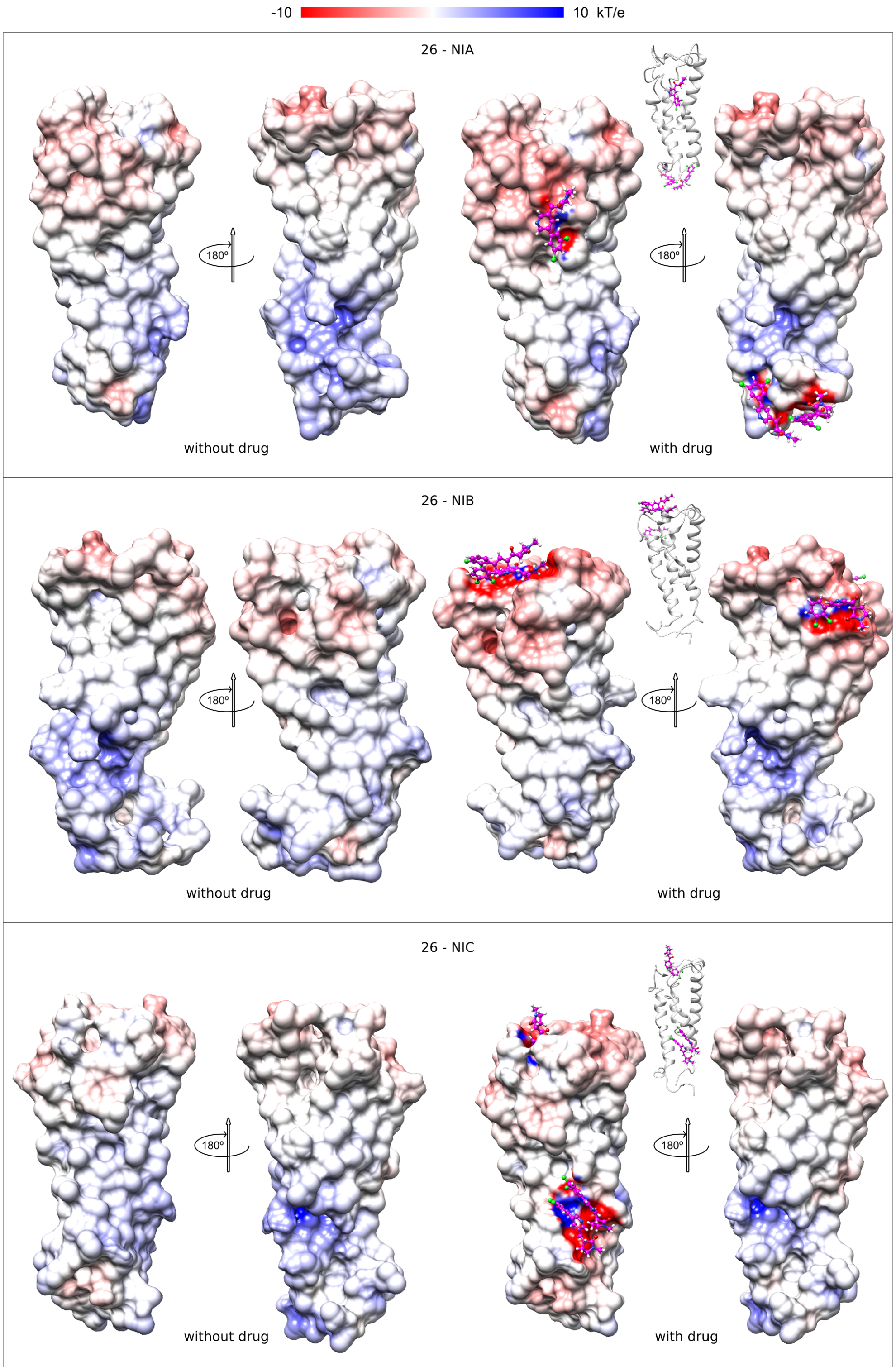

Figure S11-1. Continue next page.

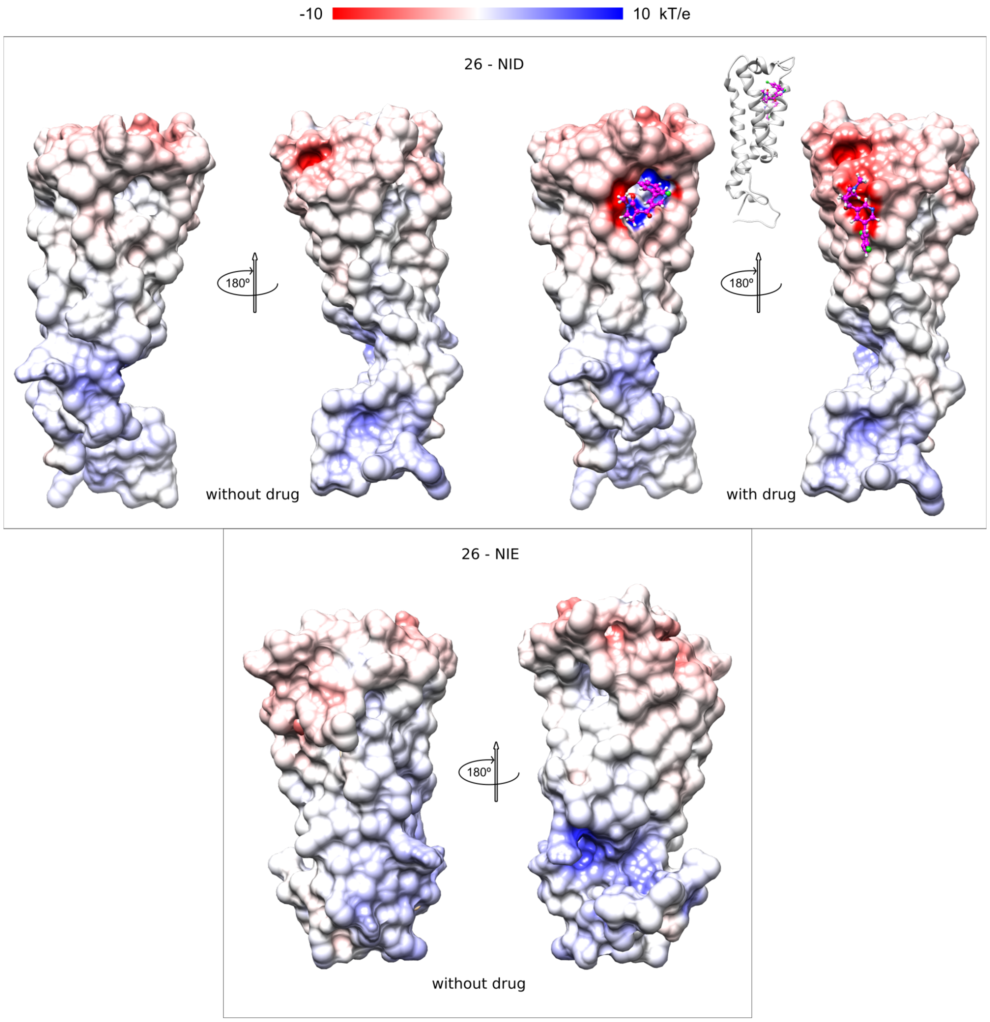

Figure S11-2. Electrostatic potential effects of NAMs on the Rv2617c surfaces in a solvated system. On the left, the protein is presented without the drug, and on the right with it. Both were rotated 180º with respect to each other to better visualize the two faces of these proteins. The color scale used was red for electronegative regions and blue for electropositive regions. The white color indicates hydrophobic areas.

Supplementary Tables

Table S1. Secondary structures conservation of the Rv1417 and Rv2617c proteins in isolation and bounded with the NAMs.

Protein	System	β-Strand	α-helix	3-10 helix	Other
	AlphaFold	22.7 (35)	40.3 (62)	1.9 (3)	35.1 (54)
	Isolate	18.8 (29)	28.6 (44)	3.9 (6)	48.7 (75)
	ASC14-NIA	20.1 (31)	24.7 (38)	3.9 (6)	51.3 (79)
**Rv1417**	ASC14-NIB	18.8 (29)	26.6 (41)	3.9 (6)	50.6 (78)
	ASC14-NIC	18.8 (29)	26.0 (40)	4.5 (7)	50.6 (78)
	ASC14-NID	17.5 (27)	26.0 (40)	5.8 (9)	50.6 (78)
	ASC14-NIE	19.5 (30)	28.6 (44)	5.8 (9)	46.1 (71)
	AlphaFold	0.0 (0)	70.5 (103)	2.7 (4)	26.7 (39)
	Isolate	0.0 (0)	44.5 (65)	5.5 (8)	50.0 (73)
	ASC26-NIA	0.0 (0)	41.8 (61)	12.3 (18)	45.9 (67)
**Rv2617**	ASC26-NIB	0.0 (0)	50.7 (74)	6.2 (9)	43.2 (63)
	ASC26-NIC	0.0 (0)	48.6 (71)	2.1 (3)	49.3 (72)
	ASC26-NID	0.0 (0)	47.9 (70)	7.5 (11)	44.5 (65)
	ASC26-NIE	0.0 (0)	62.3 (91)	2.7 (4)	34.9 (51)
	Sol14-NIA	18.8 (29)	32.5 (50)	1.3 (2)	47.4 (73)
	Sol14-NIB	18.2 (28)	28.6 (44)	1.9 (3)	51.3 (79)
**Rv1417**	Sol14-NIC	18.2 (28)	29.2 (45)	6.5 (10)	46.1 (71)
	Sol14-NID	18.8 (29)	30.5 (47)	3.9 (6)	46.8 (72)
	Sol14-NIE	20.1 (31)	30.5 (47)	5.8 (9)	43.5 (67)
	Sol26-NIA	0.0 (0)	38.4 (56)	12.3 (18)	49.3 (72)
	Sol26-NIB	0.0 (0)	46.6 (68)	8.9 (13)	44.5 (65)
**Rv2617**	Sol26-NIC	0.0 (0)	41.8 (61)	4.1 (6)	54.1 (79)
	Sol26-NID	0.0 (0)	50.0 (73)	8.2 (12)	41.8 (61)
	Sol26-NIE	0.0 (0)	56.2 (82)	6.2 (9)	37.7 (55)

The values are given as percentages and were obtained from the analysis of the structures of minimum energy obtained in the FEL analysis. The values in parentheses are the number of residues involved in the secondary structure.

Table S2. Top 10 residues that contribute to the binding free energy in the RvPs–NAMs interaction.

NAM	Active site complexes		Solvated complexes	
	Rv1417	Rv2617c	Rv1417	Rv2617c
NIA	Y22 (-37.23), L19 (-29.09), P18 (-10.21), R72 (-10.08), A23 (-9.79), R132 (-7.93), H15 (-3.20), P14 (-2.15), D9 (-1.53), F26 (-1.49)	M54 (-72.55), F114 (-69.09), Q50 (-54.41), P112 (-20.01), A57 (-19.62), N53 (-19.04), P49 (-7.77), D116 (-4.49), L46 (-4.33), T47 (-2.70)	A80 (-81.06), L61 (-48.96), V54 (-39.73), S79 (-36.15), S96 (-33.57), P5 (-29.75), G94 (-29.47), Y147 (-28.48), W95 (-28.48), D7 (-23.10)	R4 (-102.00), P5 (-79.58), S8 (-51.77), P9 (-47.90), M1 (-40.62), G45 (-38.08), S2 (-32.96), I120 (-31.38), Y119 (-29.48), V46 (-27.84)
NIB	R72 (-55.09), R85 (-35.99), R74 (-18.07), R13 (-15.89), P14 (-10.89), L88 (-10.20), P18 (-6.37), R76 (-5.72), P73 (-2.24), R91 (-1.81)	Y115 (-13.57), V109 (-9.11), I105 (-6.68), P112 (-2.65), D116 (-2.24), R133 (-2.04), F114 (-2.01), L125 (-2.00), I129 (-1.87), F122 (-1.71)	Y147 (-84.11), A80 (-73.32), I92 (-55.03), S79 (-54.18), P5 (-50.17), N6 (-41.34), D7 (-28.68), R146 (-25.28), G94 (-21.71), S96 (-18.42)	F114 (-100.43), M54 (-66.80), Y55 (-56.66), Y73 (-50.12), Q50 (-48.41), P112 (-44.30), V88 (-43.45), F42 (-40.93), I92 (-35.24), A89 (-27.48)
NIC	H15 (-40.58), R72 (-33.17), P18 (-19.01), P14 (-17.63), L19 (-16.76), R13 (-5.97), Y22 (-5.77), R132 (-2.40), R85 (-1.91), D9 (-1.91)	M54 (-36.73), Y55 (-32.18), Q50 (-16.39), F114 (-14.60), A57 (-13.68), I117 (-13.21), P112 (-11.16), L108 (-7.75), I104 (-4.66), N107 (-2.79)	R91 (-34.04), V41 (-32.07), V54 (-30.26), W109 (-29.95), K40 (-27.37), L69 (-22.53), V62 (-22.47), M123 (-22.06), A55 (-22.02), I92 (-21.44)	Q136 (-47.93), I143 (-39.49), Y55 (-34.21), M54 (-33.87), L134 (-30.50), A57 (-21.21), Q70 (-20.43), Q50 (-20.38), Y73 (-20.10), L11 (-18.82)
NID	R72 (-46.70), R74 (-32.44), R85 (-27.91), R76 (-15.08), R13 (-13.70), P14 (-8.14), W8 (-2.12), P73 (-2.00), P18 (-1.83), R91 (-1.70)	P65 (-5.22), W95 (-3.93), D62 (-2.29), L63 (-2.28), W59 (-2.25), A99 (-2.16), T6 (-2.01), H139 (-1.97), I92 (-1.95), T110 (-1.92)	I99 (-93.68), G45 (-61.66), S102 (-42.06), I92 (-40.52), Q49 (-33.39), S43 (-30.13), G94 (-30.10), A80 (-28.26), K40 (-28.25), P5 (-23.28)	W100 (-39.87), A99 (-30.47), A102 (-23.34), I78 (-19.80), R145 (-18.80), Y115 (-18.58), W95 (-18.51), L106 (-17.21), V109 (-15.09), R133 (-13.60)
NIE	R72 (-36.22), R74 (-30.23), R85 (-23.76), R13 (-13.18), P14 (-13.17), R76 (-12.81), P18 (-7.71), Y22 (-2.05), R146 (-2.02), P73 (-2.00)	P112 (-75.89), M54 (-70.19), F114 (-47.20), A57 (-40.57), N53 (-18.49), Q50 (-11.72), G111 (-9.94), P49 (-8.61), T110 (-5.71), G113 (-3.40)	I92 (-44.03), P5 (-32.55), G94 (-25.55), A80 (-19.57), V93 (-18.21), R108 (-14.12), L27 (-12.67), V128 (-12.52), F20 (-11.96), W16 (-11.93)	P112 (-147.88), M54 (-103.23), F114 (-87.86), Q50 (-69.13), A57 (-60.07), N53 (-41.86), T110 (-39.03), H51 (-38.18), V88 (-32.47), P49 (-30.08)

The numbers in parentheses are the BFE in kJ/mol measured in the last 50 ns of the MD trajectories.
